# The Role of Vascular Risk Factors in Post-Stroke Delirium: A Systematic Review and Meta-Analysis

**DOI:** 10.3390/jcm11195835

**Published:** 2022-10-01

**Authors:** Vasileios Siokas, Robert Fleischmann, Katharina Feil, Ioannis Liampas, Markus C. Kowarik, Yang Bai, Maria-Ioanna Stefanou, Sven Poli, Ulf Ziemann, Efthimios Dardiotis, Annerose Mengel

**Affiliations:** 1 Department of Neurology & Stroke, Eberhard-Karls University of Tübingen, 72076 Tübingen, Germany; 2 Hertie Institute for Clinical Brain Research, Eberhard-Karls University of Tübingen, 72076 Tübingen, Germany; 3 Department of Neurology, University Hospital of Larissa, Faculty of Medicine, School of Health Sciences, University of Thessaly, 41100 Larissa, Greece; 4 Department of Neurology, University Medicine Greifswald, 17475 Greifswald, Germany; 5 School of Basic Medical Sciences, Hangzhou Normal University, Hangzhou 310030, China

**Keywords:** stroke, delirium, vascular risk factors, PSD

## Abstract

Vascular risk factors may predispose to post-stroke delirium (PSD). A systematic review and meta-analysis were performed by searching PubMed, Web of Science, and Scopus. The primary outcome was the prevalence of vascular risk factors in PSD vs. non-PSD patients. Odds ratios (ORs) with 95% confidence intervals (CIs) and mean differences (MDs) with 95% CIs were calculated for categorical and continuous variables, respectively. Fixed effects or random effects models were used in case of low- or high-statistical heterogeneity, respectively. We found an increased prevalence of atrial fibrillation (OR = 1.74, *p* = 0.0004), prior stroke (OR = 1.48, *p* < 0.00001), coronary artery disease (OR = 1.48, *p* < 0.00001), heart failure (OR = 2.01, *p* < 0.0001), and peripheral vascular disease (OR = 2.03, *p* < 0.00001) in patients with vs. without PSD. PSD patients were older (MD = 5.27 y, *p* < 0.00001) compared with their non-PSD counterparts. Advanced age, atrial fibrillation, prior stroke, coronary artery disease, heart failure, and peripheral vascular disease appeared to be significantly associated with PSD.

## 1. Introduction

Post-stroke delirium (PSD) comprises a frequent, severe, and yet potentially preventable complication of acute strokes [1,2,3]. Although PSD typically occurs within the first few days (3–7 days) from acute stroke onset [4,5], a delayed manifestation has also been reported [4,5]. PSD typically presents with a fluctuating course, and thus it may be underrecognized; consequently, in severe cases, it may rapidly progress into a non-reversible condition [6].

Recent meta-analyses have highlighted the burden caused by PSD, and reported that almost 25% of acute stroke patients develop PSD, whereas there are other studies reporting a PSD rate as high as 60% [7]. In terms of clinical prognosis, PSD has been linked to unfavorable short- and long-term outcomes [8], including increased inpatient mortality (i.e., almost four-fold higher in PSD compared with non-PSD patients), prolonged hospital stays (i.e., on average 10 days longer in PSD patients), poorer functional status, reduced independency at discharge (i.e., more likely to be discharged to a nursing unit), and increased 1-year mortality [8]. 

In clinical practice, currently available PSD treatment options are characterized by limited efficacy [9,10]. Increasing interest has thus been recently drawn towards the identification of reliable risk factors that importantly may facilitate early recognition and PSD prevention, because the pathophysiology of PSD remains to date poorly elucidated, and biomarkers are unavailable [11]. Unfortunately, there is still conflicting evidence in the literature regarding PSD risk factors [12,13].

Vascular risk factors have been previously linked to PSD [14]; however, earlier studies have yielded inconsistent results [15,16,17]. In addition, previous meta-analyses have focused on the role of few specific risk factors, including stroke localization and pre-existing cognitive decline in PSD [7,8,18,19,20,21]. While narrative and scoping reviews regarding the main predisposing vascular factors of PSD have been published [17,22], to the best of our knowledge, the association between vascular risk factors and PSD has not been previously assessed using a meta-analytical approach.

In view of this gap, a systematic review of the literature, comprising all of the so-far published literature on vascular risk factors and PSD, was performed. We then aimed to increase the class evidence of the associations between vascular risk factors and PSD through an aggregate data meta-analysis, to assess associations between vascular risk factors and PSD.

## 2. Materials and Methods

### 2.1. General Information

For the current meta-analysis, we applied the Preferred Reporting Items Systematic Reviews and Meta-Analyses (PRISMA) guidelines (File S1) [23]. This study was not registered in any database. The systematic review and meta-analysis were independently performed by two authors (V.S. and I.L.). Disagreements were resolved after discussion with a third tie-breaking evaluator (A.M.). This study did not require an ethical board approval or written informed consent by the patients according to the study design (systematic review and meta-analysis).

### 2.2. Literature Search Strategy and DATA Source

We conducted a database search in the PubMed, Web of Science, and Scopus databases for eligible articles presenting data regarding the risk factors for PSD. The last literature search was performed on 28 April 2022. We used the term “stroke” in combination with the term “delirium”. The complete PubMed algorithm of the conducted literature review search for the current meta-analysis was (“stroke”[MeSH Terms] OR “stroke”[All Fields] OR “strokes”[All Fields] OR “stroke’ s”[All Fields]) AND (“delirium”[MeSH Terms] OR “delirium”[All Fields] OR “deliriums”[All Fields]). We identified and removed the duplicates using EndNote citation manager software (v20, Clarivate, Philadelphia, PA, USA).

### 2.3. Identification of Eligible Articles and DATA Source

The literature was screened for study suitability based on title and abstract. Only subject-relevant studies were therefore considered as eligible. We retrieved the full texts from the articles that passed the initial screening. Furthermore, the reference lists of the included articles (see inclusion criteria below) were screened for further eligible studies that may have been missed from the initial scan.

### 2.4. Inclusion/Exclusion Criteria

Studies were included in the meta-analysis according to the following criteria:

(1) They were published before 28 April 2022; (2) they presented data regarding the risk factors of interest for the two groups (PSD vs. non-PSD) or for the overall sample and at least for one subgroup (PSD or non-PSD); (3) respective data should be presented as absolute numbers or proportions for categorical variables and as means [with the standard deviations (SD)] or median [with the respective range or interquartile range (IQR)] for continuous variables; (4) no limitations were implemented with respect to study design (cross-sectional, case-control, or cohort studies with prospective or retrospective data collection were retrieved); (5) delirium detection was either routinely and prospectively performed by attending physicians or via the capitalization of secure, primary hospital records, using standard assessment protocols in both cases; and (6) the studies were written in the English language. In case of multiple articles presenting results from the same sample, data from the article with the largest sample or the one presenting the respective relative data were included. 

Studies were excluded according to the following criteria:

(1) reviews, meta-analyses, and case-reports; (2) studies not involving human subjects; (3) studies with full texts and abstracts not published in English; (4) book chapters, conference abstracts, and posters presentations; (5) studies without (or with infeasible extraction of) relevant data for both groups (PSD vs. non-PSD); (6) studies implementing diagnostic procedures not based on direct (attending physician) or primary medical records assessments (e.g., International Classification of Diseases (ICD)-based PSD establishment, self-reported diagnosis, or surrogate-reported diagnosis); and (7) retracted articles.

### 2.5. Quality Assessment Tool

We applied the Newcastle–Ottawa scale (NOS) for case-control and cohort studies and the modified NOS for cross-sectional studies in order to assess the quality of each study, as previously described (Table S1) [24,25]. 

### 2.6. Data Extraction

When possible, we extracted the following data from each eligible study: (1) authors, (2) year of publication, (3) ethnicity/location of study, (4) numbers (n) of PSD and non-PSD cases, (5) phenotypes of the included participants [ischemic stroke, intracerebral hemorrhage (ICH), transient ischemic attack (TIA), subarachnoid hemorrhage (SAH)], (6) age, (7) sex distribution, (8) method of PSD assessment, and (9) times of PSD assessments. Moreover, we extracted data regarding histories of hypertension, smoking, alcohol consumption/abuse, previous or current atrial fibrillation (AF), diabetes mellitus (DM), hyperlipidemia, prior stroke (also including TIA), coronary artery disease (CAD) including also myocardial infraction, ischemic heart disease, and atherosclerotic heart disease, heart failure (HF), and peripheral vascular (PVD). The mean value [with the respective SD] or the median value (with either the range or IQR) of the continuous variables (age) and the total number (n) of the categorical variables (history of hypertension, smoking, alcohol consumption/abuse, previous or current AF, DM, hyperlipidemia, prior stroke, CAD, HF, and PVD) were obtained. We applied the method of Hozo et al. (2005), to calculate the mean and SD when the median and range were provided [26]. Moreover, we used the method of Wan et al. (2014), to calculate mean and SD when the median and range or IQR were provided, after checking for normal distribution [27]. Means and SDs from ≥2 groups (e.g., PSD and non-PSD) were combined following the recommended formula by the *Cochrane Handbook for Systematic Reviews* [28].

### 2.7. Statistical Analysis

#### 2.7.1. Calculation of the Effect Size

Statistical analyses were performed using the Review Manager (RevMan) Version 5.4.1 (https://training.cochrane.org/online-learning/core-software/revman/revman-5-download, accessed on 15 December 2021). The odds ratios (ORs) and the respective 95% confidence intervals (CIs) were calculated in order to estimate the effect of the categorical variables (1. male sex, 2. hypertension, 3. smoking, 4. alcohol consumption/abuse, 5. previous or current AF, 6. DM, 7. hyperlipidemia, 8. prior stroke, 9. CAD, 10. HF, and 11. PVD) on PSD. We estimated the mean differences (MDs) and their respective 95% CIs for the continuous variable (age). The inverse variance method of individual effects was used. An alpha error of 5% (two-tailed *p* < 0.05) was established as the statistical significance threshold. In case of an association between a risk factor and an outcome, we performed further subgroup analyses, based on the type of stroke. Two groups were created: 1. Ischemic stroke/TIA and 2. ICH/SAH, whenever possible.

#### 2.7.2. Assessment of Heterogeneity

The heterogeneity was calculated with the Cochran’s Q and I^2^ tests [29,30]. We defined as high-heterogeneity values P_Q_ < 0.10 and/or I^2^ > 75% [31]. In case of high heterogeneity, the random effects (RE) model was used; in case of low heterogeneity, we used the fixed effects (FE) model [32].

#### 2.7.3. Publication Bias and Sensitivity Analyses

The publication bias was graphically assessed by means of Funnel plot asymmetry. We performed sensitivity analyses by omitting one study at a time, aiming to calculate the effect of each individual study.

## 3. Results

### 3.1. Study Selection and Study Characteristics

**Figure 1:**
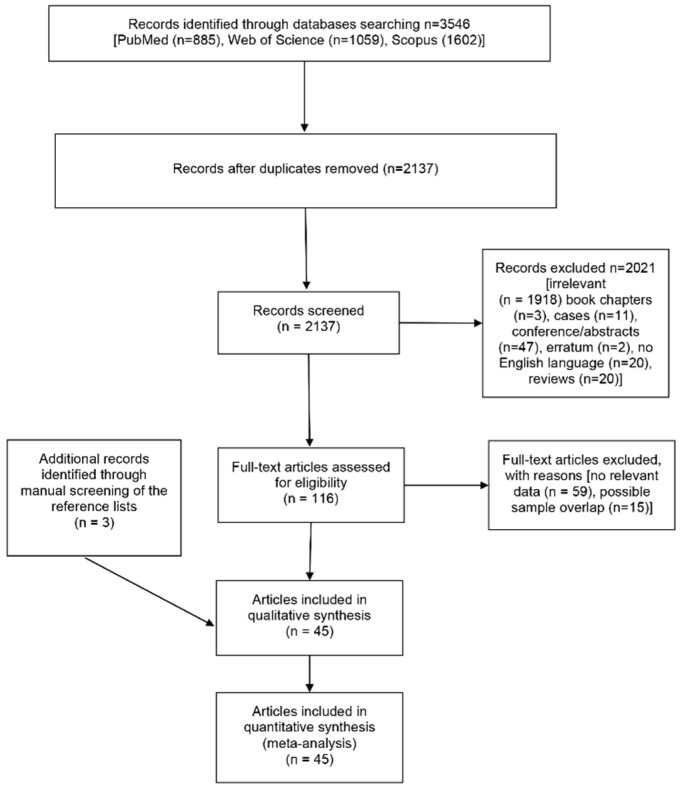
The flowchart presenting the selection procedure of eligible studies.

The structured literature search yielded 3546 entries (885 from PubMed, 1059 from Web of Science, and 1602 from Scopus). After the removal of duplicates, 2137 entries were scanned. Finally, 116 full texts were assessed for eligibility, with 42 articles [5,6,10,33,34,35,36,37,38,39,40,41,42,43,44,45,46,47,48,49,50,51,52,53,54,55,56,57,58,59,60,61,62,63,64,65,66,67,68,69,70,71] fulfilling all criteria. Three additional studies [59,72,73] were identified through reference lists, leading to a total of 45 articles that were included in the current meta-analysis. Study characteristics are accessible in Table S2. The flowchart presenting the literature review search is presented in Figure 1.

### 3.2. Tests of Heterogeneity, Effect Size and Publication Bias 

#### 3.2.1. Age

Thirty studies (8875 subjects) were included in this analysis. For the main analysis, we used the hypoactive (largest sample) type of delirium of the study of Fialho Silva et al. (2021). High heterogeneity (P_Q_ < 0.00001, I^2^ = 80%) was revealed. Statistically significant higher age was observed in patients with PSD compared with those without PSD (RE: MD = 5.27 y, 95% CI = (4.41, 6.14), *p* < 0.00001) (Figure S1a). Sensitivity analyses by omitting each one study at a time provided similarly significant differences (data not shown). The analysis for publication bias revealed no significant asymmetry (Figure S2a). The values for the study of Henon et al. (1999) were calculated using the formula of Hozo et al. (2005), and values for the studies of Alvarez-Perez et al. (2017) and Shaw et al. (2019) were calculated using the formula of Wan et al. (2014), as previously described. The sensitivity analysis including either the hyperactive [P_Q_ < 0.00001, I^2^ = 80%, (RE: MD = 5.21y, 95% CI = (4.34, 6.08), *p* < 0.00001] or mixed [P_Q_ < 0.00001, I^2^ = 80%, (RE: MD = 5.29y, 95% CI = (4.42, 6.16), *p* < 0.00001] type of delirium of the study of Fialho Silva et al. (2021) did not change the results.

#### 3.2.2. Sex

Thirty-six studies (10,672 subjects) were included in this analysis. The analysis revealed high heterogeneity (P_Q_ = 0.07, I^2^ = 28%). No statistically significant difference for the effect of male sex was observed between patients with PSD and those without PSD (RE: OR = 0.98, 95% CI = (0.87, 1.11), *p* = 0.79) (Figure S1b). Sensitivity analyses by omitting each one study at a time similarly provided no significant differences (data not shown). The analysis for publication bias revealed no significant asymmetry, with high standard error (SE) for the study of Shih et al. (2007) (Figure S2b).

#### 3.2.3. Hypertension

Twenty-three studies (7293 subjects) were included in this analysis, while no significant heterogeneity (P_Q_ = 0.45, I^2^ = 0%) was observed. There was no statistically significant difference regarding hypertension between patients with PSD and those without PSD [FE: OR = 1.12, 95% CI = (0.99, 1.28), *p* = 0.07] (Figure S1c). Sensitivity analyses by omitting each one study at a time similarly provided no significant differences (data not shown), with the exceptions of the removal of Gustafsson et al. (1991) [FE: OR = 1.15, 95% CI = (1.01, 1.31), *p* = 0.04], Melkas et al. (2011) [FE: OR = 1.14, 95% CI = (1.01, 1.30), *p* = 0.04], Alvarez-Perez et al. (2017) [FE: OR = 1.15, 95% CI = (1.00, 1.31), *p* = 0.04], and Hoyosa et al. (2018) [FE: OR = 1.15, 95% CI = (1.01, 1.31), *p* = 0.03], where statistically significant differences were revealed. This can be explained by the fact that these studies reported a lower prevalence of hypertension in the PSD group compared with the non-PSD. The analysis for publication bias revealed no significant asymmetry (Figure S2c).

#### 3.2.4. Alcohol

Fourteen studies (4402 subjects) were included in this analysis, while no significant heterogeneity (P_Q_ = 0.21, I^2^ = 23%) was observed. There was no statistically significant difference regarding alcohol consumption/abuse between patients with PSD and those without PSD [FE: OR = 1.19, 95% CI = (0.97, 1.47), *p* = 0.10] (Figure S1d). For the analysis of the alcohol consumption, a simplification was made. We included studies independently of the amount and the frequency of the alcohol consumption. However, the sensitivity analyses by omitting each one study at a time similarly provided no significant differences (data not shown), with the exceptions of the removals of Caeiro et al. (2004) [FE: OR = 1.26, 95% CI = (1.01, 1.56), *p* = 0.04] and Alvarez-Perez et al. (2017) [FE: OR = 1.29, 95% CI = (1.01, 1.64), *p* = 0.04], where statistically significant differences were revealed. These studies reported a lower prevalence of alcohol consumption at the PSD group compared with the non-PSD. The analysis for publication bias revealed no significant asymmetry, with the study of Ng. et al. (2019) showing a large SE (Figure S2d).

#### 3.2.5. Smoking

Fourteen studies (4566 subjects) were included in this analysis, while significant heterogeneity (P_Q_ = 0.06, I^2^ = 39%) was observed. There was no statistically significant difference regarding smoking between patients with PSD and those without PSD [RE: OR = 0.94, 95% CI = (0.73, 1.21), *p* = 0.62] (Figure S1e). Sensitivity analyses by omitting each one study at a time similarly provided no significant differences (data not shown). The analysis for publication bias revealed no significant asymmetry (Figure S2e).

#### 3.2.6. DM

Twenty-six studies (7754 subjects) were included in this analysis. No significant heterogeneity (P_Q_ = 0.14, I^2^ = 24%) was observed. There was no statistically significant difference regarding smoking between patients with PSD and those without PSD [FE: OR = 1.10, 95% CI = (0.96, 1.26), *p* = 0.15] (Figure S1f). Sensitivity analyses by omitting each one study at a time similarly provided no significant differences (data not shown). The analysis for publication bias revealed no significant asymmetry (Figure S2f). 

#### 3.2.7. AF

Thirteen studies (5016 subjects) were included in this analysis. Significant heterogeneity (P_Q_ < 0.0001, I^2^ = 70%) was observed. There was a statistically significant difference regarding AF between patients with PSD and those without PSD [RE: OR = 1.74, 95% CI = (1.28, 2.36), *p* = 0.0004] (Figure S1g). Sensitivity analyses by omitting each one study at a time similarly provided significant differences (data not shown). The analysis for publication bias revealed no significant asymmetry (Figure S2g).

#### 3.2.8. Hyperlipidemia

Eight studies (1375 subjects) were included in this analysis. Regarding the study of Shish et al. (2007), we included in the main analysis the lowest estimation sample (meaning the lower number of patients with hypercholesterolemia and/or hypertriglyceridemia) in order to avoid any overlap. Significant heterogeneity (P_Q_ = 0.62, I^2^ = 0%) was observed. There was no statistically significant difference regarding hyperlipidemia between patients with PSD and those without PSD [FE: OR = 0.99, 95% CI = (0.74, 1.30), *p* = 0.92] (Figure S1h). Sensitivity analyses by omitting each one study at a time similarly provided no significant differences (data not shown). The analysis for publication bias revealed no significant asymmetry (Figure S2h).

#### 3.2.9. Prior Stroke 

Twenty-one studies (6670 subjects) were included in this analysis. For the studies of Gustafson et al. (1991), Sheng et al. (2006), Kara et al. (2013), Kotfis et al. (2018), Zaitun et al. (2019), and Pendlebury et al. (2022), we included in the main analysis the lower estimation sample (meaning the lower number of patients with stroke and/or TIA) in order to avoid any overlap. No significant heterogeneity (P_Q_ = 0.61, I^2^ = 0%) was observed. There was a statistically significant difference regarding the previous cerebral adverse event between patients with PSD and those without PSD [FE: OR = 1.48, 95% CI = (1.28, 1.70), *p* < 0.00001] (Figure S1i). Sensitivity analyses by omitting each one study at a time similarly provided significant differences (data not shown). The analysis for publication bias revealed no significant asymmetry (Figure S2i). 

#### 3.2.10. CAD

Twelve studies (4966 subjects) were included in this analysis. Regarding the study of Kotfis et al. (2018), we included in the main analysis the lowest estimation sample (meaning the lower number of patients with ischemic heart disease and/or myocardial infraction) in order to avoid any sample overlap. No significant heterogeneity (P_Q_ = 0.20, I^2^ = 25%) was observed. There was a statistically significant difference regarding CAD between patients with PSD and those without PSD [FE: OR = 1.48, 95% CI = (1.25, 1.76), *p* < 0.00001] (Figure S1j). Sensitivity analyses by omitting each one study at a time similarly provided significant differences (data not shown). The analysis for publication bias revealed no significant asymmetry (Figure S2j).

#### 3.2.11. HF

Five studies (1299 subjects) were included in this analysis. No significant heterogeneity (P_Q_ = 0.41, I^2^ = 0%) was observed. There was a statistically significant difference regarding HF between patients with PSD and those without PSD [FE: OR = 2.01, 95% CI = (1.44, 2.79), *p* < 0.0001] (Figure S1k). In the sensitivity analyses, the significance did not remain after the removal of the study of Kotfis et al. (2019) [FE: OR = 1.57, 95% CI = (0.88, 2.81), *p* = 0.33] (data not shown). The analysis for publication bias revealed no significant asymmetry (Figure S2k).

#### 3.2.12. PVD

Four studies (1660 subjects) were included in this analysis. No significant heterogeneity (P_Q_ = 0.38, I^2^ = 3%) was observed. There was a statistically significant difference regarding PVD between patients with PSD and those without PSD [FE: OR = 2.03, 95% CI = (1.53, 2.70), *p* < 0.00001] (Figure S1l). Sensitivity analyses by omitting each one study at a time similarly provided significant differences, with the exception of the removal of Kotfis et al. (2018) [FE: OR = 1.65, 95% CI = (0.99, 2.77), *p* = 0.06], where the significance did not remain. The analysis for publication bias revealed no significant asymmetry (Figure S2l). Summary of results of the current meta-analysis for are the vascular factors are presented at Table 1.

### 3.3. Subgroup Analysis Based on the Type of Stroke 

Subgroup analyses based on the stroke type (1. Ischemic stroke/TIA and 2. ICH/SAH) were possible for the significantly associated risk factors (age, AF, prior stroke, CAD, HF, and PVD) for both of the subgroups, with exception of the HF and the PDV for the ICH/SAH analysis. Statistical significance was maintained in the ischemic stroke/TIA group for the six out of seven examined risk factors, namely AF [FE: OR = 2.47, 95% CI = (1.83, 3.33), *p* < 0.00001], prior stroke [FE: OR = 1.81, 95% CI = (1.41, 2.33), *p* < 0.00001], age [RE: MD = 5.16y, 95% CI = (3.73, 6.58), *p* < 0.00001], HF [FE: MD = 1.98, 95% CI = (1.40, 2.81), *p* = 0.001], and the PVD [FE: MD = 2.00, 95% CI = (1.48, 2.68), *p* < 0.00001]. The statistical significance was lost for the CAD [RE: MD = 1.22, 95% CI = (0.74, 2.01), *p* = 0.45] (Figure S3a to Figure S3f). Respective results are presented at Table 2.

Statistical significance was maintained for the ICH group, with the exception of AF, where only a marginal trend for association was found. Namely, AF [FE: OR = 0.70, 95% CI = (0.50, 1.00), *p* = 0.05], prior stroke [FE: OR = 1.67, 95% CI = (1.16, 2.39), *p* = 0.005], age [RE: MD = 2.70y, 95% CI = (0.94, 4.45), *p* = 0.003], and CAD [FE: OR = 1.85, 95% CI = (1.24, 2.76), *p* = 0.002] (Figures S4a–S4d). In fact, AF was less frequent in ICH patients who developed PSD. Respective results are presented at Table 3.

## 4. Discussion

In the present systematic review and meta-analysis, we examined whether vascular risk factors, including age, male sex, smoking, alcohol, hypertension, DM, AF, hyperlipidemia, prior stroke, CAD, HF, and PVD are associated with PSD. Our analyses reveal an increased prevalence of AF, prior stroke, CAD, HF, and PVD in patients with PSD compared with their non-PSD counterparts. These associations retained statistical significance in the subgroup analyses based on stroke subtype (ischemic stroke/TIA and ICH/SAH), with the exception of CAD in the ischemic stroke group and AF in the ICH group. Conversely, hypertension and alcohol consumption did not appear to be associated with PSD, because associations were only evident in a subset of the sensitivity analyses and not in the main analyses. These findings highlight that AF, prior stroke, CAD, HF, and PVD may predispose to PSD, and provide a rationale for the selection of predictors that need to be considered for prospective studies validating a multivariable predictive model for PSD. However, some further considerations should be taken into account for an accurate interpretation of the present findings.

In our analysis, AF was almost two-fold more prevalent in patients with PSD. This is of particular interest, considering that AF is the most common cardiac arrhythmia [74] and that the vast majority of patients with AF belong to the age group over 65 y, a group that usually suffers from vascular pathology and is at greater risk for embolic stroke [12]. However, as the PSD group was also older compared with the non-PSD group, a net causal link between AF and PSD cannot be inferred with absolute certainty. The association between AF and PSD remained significant in the subgroup analysis of the ischemic stroke/TIA patients, with a trend towards an opposite association in the ICH subgroup. AF is a common cause of thromboembolism [75]. Emboli arising from the cardiac chambers are usually large, and as such may lead to a severe ischemic stroke with the involvement of multiple cerebral vascular territories [76,77]. Consequently, it may be related with greater impact on brain networks, leading to defective brain connectivity and alterations in brain dynamics that potentially relate to PSD [78].

Based on our meta-analyses, the prevalence of CAD was almost 1.5-fold higher in PSD patients. The frequency of delirium among patients with CAD appears to be lower compared with those with stroke [34,79,80]. Coronary artery bypass grafting (CABG) is a common factor for a post-operative delirium [81]. Moreover, post-operative delirium after a CABG is more frequent in patients with compared with those without covert stroke [82]. Consequently, CAD may constitute an additive risk for delirium in stroke patients compared with non-stroke patients. It would be important that future studies could also focus on differences between delirium characteristics in stroke patients who underwent an operative procedure and those who did not. 

Our analyses revealed that HF and PVD were two-times more frequent in PSD patients. However, these results derived from a relatively small number of studies (five and four, respectively). Moreover, the ORs follow that of the most-weighted study (Kotfis et al. 2019 for the HF and Kotfis et al. 2018 for the PVD), the exclusion of which in the sensitivity analysis led to no-longer statistically significant results. Finally, as HF and PVD have also been reported as risk factors for delirium in non-stroke patients [83,84], further studies are needed for a robust generalizability of the role of these two factors in PSD. 

We also found that patients with PSD were 5.27 years older compared with non-PSD patients. Age is a non-modifiable risk factors, and there is a great amount of evidence of its contribution to delirium [85]. Moreover, older age may increase precipitating delirium factors (e.g., infections and hydroelectrolytic disorders) [86], denoting that the control of modifiable factors as much as possible is likely of great importance to prevent PSD. 

Prior stroke was associated with the risk of developing PSD. This may be explained by the fact that prior stroke may lead to greater clinical impairment and thus to increased complications (such as aspiration pneumonia or immobilization), which could also lead to delirium [48,50,87]. 

There are additional factors, apart from those examined in our meta-analysis, that possibly contribute to the development of PSD. Pre-existing cognitive decline/dementia have been reported to attribute an almost four-fold higher risk for PSD [19]. Moreover, the stroke location appears to be an additional factor, as delirium has been reported to be more common in stroke patients with cortical, supratentorial, and anterior circulation lesions [20]. 

A better understanding of the predisposing factors leading to PSD could be beneficial for the stroke’s overall cost-of-illness. Indeed, PSD is related to a greater overall cost of hospitalization, though without being attributed to a single factor [69]. Moreover, a deeper knowledge of PSD’s predisposing factors may provide physicians with more personalized approaches regarding prevention and treatment aiming at improving functional short- and long-term outcomes after PSD. 

Among the strengths of our study is the extended literature search, obtaining a comprehensive summary of the published literature evaluating PSD and risk factors. Moreover, we performed sensitivity analyses, providing additional robustness to our conclusions. At this point, the limitations of this meta-analysis must also be acknowledged. Firstly, in an attempt to maximize the study population, we included studies irrespective of their design, the time of delirium assessment, and the delirium type. Moreover, there was heterogeneity in the included examined phenotypes, as few studies presented merged data by combining different stroke sub-phenotypes. Thus, in the main analysis we present the effects of the risk factors in a combined phenotype consisting of patients with ischemic stroke, ICH, TIA and SAH, with additional subgroup analyses based on the type of stroke in case of a significant association of the examined risk factor with PSD. Moving on, we focused only on predisposing vascular risk factors and stroke severity, as we did not meta-analyze other potential co-factors (e.g., infections, drugs, or clinical presentation, among others). Furthermore, we meta-analyzed aggregate data and, thus, the reported associations cannot be adjusted for subjects’ baseline characteristics and potential PSD confounders. Finally, though unlikely, the possibility that some eligible studies failed to be identified cannot be completely ruled out. 

## 5. Conclusions

In conclusion, with the current meta-analysis, we provide quantitative data that suggest the increased prevalence and possible causal-contributions of age, AF, prior stroke, CAD, HF, and PVD to PSD development. Considering the as-of-yet limited and often ineffective therapeutic approaches for PSD, it will be of interest to observe to what extent a personalized prevention and treatment of the modifiable PSD vascular risk factors could be effectively applied to lower the occurrence of PSD. Towards this direction, further collaborative and multiethnic studies are needed in order for PSD to be more effectively prevented and managed.

## Figures and Tables

**Table 1:** Summary of results of the current meta-analysis.

Risk Factor	Number of Studies	Number of Subjects	Heterogeneity		Model	Effect SIZE	*p*-Value
			P_Q_	I^2^			
Age	30	8875	<0.00001	80%	RE	5.27 [4.41, 6.14] *	**<0.00001**
Sex	36	10,672	0.07	28%	RE	0.98 [0.87, 1.11] ^	0.79
Hypertension	23	7293	0.45	0%	FE	1.12 [0.99, 1.28] ^	0.07
Alcohol	14	4402	0.21	23%	FE	1.19 [0.97, 1.47] ^	0.10
Smoking	14	4566	0.06	39%	RE	0.94 [0.73, 1.21] ^	0.62
DM	26	7754	0.14	24%	FE	1.10 [0.96, 1.26] ^	0.15
AF	13	5016	<0.0001	70%	RE	**1.74 [1.28, 2.36] ^**	**0.0004**
Hyperlipidemia	8	1375	0.62	0%	FE	0.99 [0.74, 1.30] ^	0.92
Prior stroke	21	6770	0.61	0%	FE	**1.48 [1.28, 1.70] ^**	**<0.00001**
CAD	12	4966	0.20	25%	FE	**1.48 [1.25, 1.76] ^**	**<0.00001**
HF	5	1299	0.41	0%	FE	**2.01 [1.44, 2.79] ^**	**<0.0001**
PVD	4	1660	0.38	3%	FE	**2.03 [1.53, 2.70] ^**	**<0.00001**

* mean difference, with 95% CI; ^ odds ratio, with 95% CI; DM, diabetes mellitus; AF, atrial fibrillation; CAD, coronary artery disease; HF, heart failure; PVD, peripheral vascular disease; Statistically significant values are given in bold.

**Table 2:** Results of the current meta-analysis for the ischemic stroke/TIA patients.

Risk Factor	Number of Studies	Number of Subjects	Heterogeneity		Model	Effect Size	*p*-Value
			P_Q_	I^2^			
Age	12	3311	0.0002	69%	RE	5.16 [3.73, 6.58] *	**<0.00001**
AF	3	1324	0.34	7%	FE	**2.47 [1.83, 3.33] ^**	**<0.00001**
Prior stroke	4	2056	0.19	38%	FE	**1.81 [1.41, 2.33] ^**	**<0.00001**
CAD	5	1798	0.06	55%	RE	1.22 [0.74, 2.01] ^	0.45
HF	3	1083	0.15	47%	FE	**1.98 [1.40, 2.81] ^**	**0.0001**
PVD	3	1458	0.23	32%	FE	**2.00 [1.48, 2.68] ^**	**<0.00001**

* mean difference, with 95% CI; ^ odds ratio, with 95% CI; TIA, transient ischemic attack; AF, atrial fibrillation; NIHSS, National Institutes of Health Stroke Scale; CAD, coronary artery disease; HF, heart failure; PVD, peripheral vascular disease; Statistically significant values are given in bold.

**Table 3:** Results of the current meta-analysis for the patients with ICH and/or SAH.

Risk Factor	Number of Studies	Number of Subjects	Heterogeneity		Model	Effect Size	*p*-Value
			P_Q_	I^2^			
Age	4	1127	0.48	0%	RE	2.70 [0.94, 4.45] *	**0.003**
AF	2	838	0.96	0%	FE	0.70 [0.50, 1.00] ^	0.05
Prior stroke	2	838	0.62	0%	FE	**1.67 [1.16, 2.39] ^**	**0.005**
CAD	2	838	0.53	0%	FE	**1.85 [1.24, 2.76] ^**	**0.002**

* mean difference, with 95% CI; ^ odds ratio, with 95% CI; ICH, intracerebral hemorrhage; SAH, subarachnoid hemorrhage; AF, atrial fibrillation; CAD, coronary artery disease; statistically significant values are given in bold.

## Data Availability

Not applicable.

## Supplementary Materials

The following supporting information can be downloaded at: https://www.mdpi.com/article/10.3390/jcm11195835/s1, File S1. PRISMA 2020 checklist; Table S1. Quality evaluation according to the Newcastle–Ottawa Scale (NOS—for case-control and cohort studies) and modified NOS (for cross-sectional studies); Table S2. Characteristics of the included studies; Figures S1a to S1l. The Forest plots presenting the results from the meta-analysis of vascular risk factors and PSD; Figure S1a. The Forest plot presenting the results from the meta-analysis of age and PSD; Figure S1b. The Forest plot presenting the results from the meta-analysis of sex and PSD; Figure S1c. The Forest plot presenting the results from the meta-analysis of hypertension and PSD; Figure S1d. The Forest plot presenting the results from the meta-analysis of alcohol and PSD; Figure S1e. The Forest plot presenting the results from the meta-analysis of smoking and PSD; Figure S1f. The Forest plot presenting the results from the meta-analysis of DM and PSD; Figure S1g. The Forest plot presenting the results from the meta-analysis of AF and PSD; Figure S1h. The Forest plot presenting the results from the meta-analysis of hyperlipidemia and PSD; Figure S1i. The Forest plot presenting the results from the meta-analysis of prior stroke and PSD; Figure S1j. The Forest plot presenting the results from the meta-analysis of CAD and PSD; Figure S1k. The Forest plot presenting the results from the meta-analysis of HF and PSD; Figure S1l. The Forest plot presenting the results from the meta-analysis of PVD and PSD; Figures S2a to S2l. The Funnel plots presenting the results from the meta-analysis of vascular risk factors and PSD; Figure S2a. The Funnel plot presenting the results from the meta-analysis of age and PSD; Figure S2b. The Funnel plot presenting the results from the meta-analysis of sex and PSD; Figure S2c. The Funnel plot presenting the results from the meta-analysis of hypertension and PSD; Figure S2d. The Funnel plot presenting the results from the meta-analysis of alcohol and PSD; Figure S2e. The Funnel plot presenting the results from the meta-analysis of smoking and PSD; Figure S2f. The Funnel plot presenting the results from the meta-analysis of DM and PSD; Figure S2g. The Funnel plot presenting the results from the meta-analysis of AF and PSD; Figure S2h. The Funnel lot presenting the results from the meta-analysis of hyperlipidemia and PSD; Figure S2i. The Funnel plot presenting the results from the meta-analysis of prior stroke and PSD; Figure S2j. The Funnel plot presenting the results from the meta-analysis of CAD and PSD; Figure S2k. The Funnel plot presenting the results from the meta-analysis of HF and PSD; Figure S2l. The Funnel plot presenting the results from the meta-analysis of PVD and PSD; Figures S3a to S3f. The Forest plots presenting the results from the meta-analysis of age, AF, prior stroke, CAD, HF, PVD, and PSD in the stroke/TIA subgroup; Figure S3a. The Forest plot presenting the results from the meta-analysis of age and PSD in the stroke/TIA subgroup; Figure S3b. The Forest plot presenting the results from the meta-analysis of AF and PSD in the stroke/TIA subgroup; Figure S3c. The Forest plot presenting the results from the meta-analysis of prior stroke and PSD in the stroke/TIA subgroup; Figure S3d. The Forest plot presenting the results from the meta-analysis of CAD and PSD in the stroke/TIA subgroup; Figure S3e. The Forest plot presenting the results from the meta-analysis of HF and PSD in the stroke/TIA subgroup; Figure S3f. The Forest plot presenting the results from the meta-analysis of PVD and PSD in the stroke/TIA subgroup; Figures S4a to S4d. The Forest plots presenting the results from the meta-analysis of age, AF, prior stroke, CAD, and PSD in the ICH and/or SAH subgroup; Figure S4a. The Forest plot presenting the results from the meta-analysis of age and PSD in the ICH and/or SAH subgroup; Figure S4b. The Forest plot presenting the results from the meta-analysis of AF and PSD in the ICH and/or SAH subgroup; Figure S4c. The Forest plot presenting the results from the meta-analysis of prior stroke and PSD in the ICH and/or SAH subgroup; Figure S3d. The Forest plot presenting the results from the meta-analysis of CAD and PSD in the ICH and/or SAH subgroup.
