# Supplementary material for: The Role of Vascular Risk Factors in Post-Stroke Delirium: A Systematic Review and Meta-Analysis
Source: J Clin Med. 2022 Oct 1;11(19):5835. doi: 10.3390/jcm11195835 (PMC9571874; doi:10.3390/jcm11195835)
Supplement: Supplementary file 1 [file jcm-11-05835-s001.zip › Figures S1a to S1l.pdf]

Figures S1a to S1l. The Forest Plots presenting the results from meta-analysis of vascular risk factors and PSD

Study or Subgroup	Delirium Mean	Delirium SD	Delirium Total	No Delirium Mean	No Delirium SD	No Delirium Total	Weight	Mean Difference IV, Random, 95% CI
Nicolai & Lazzarino (1994)	73.6	4.9	13	72.1	3.5	20	3.4%	1.50 [-1.57, 4.57]
Henon et al. (1999)	78	12.75	49	74	9.83	153	2.7%	4.00 [0.11, 7.89]
Sheng et al. (2006)	81.5	5.5	39	78.4	6.9	117	4.4%	3.10 [0.97, 5.23]
Shih et al. (2007)	66.3	13.05	14	64.8	9.85	15	0.9%	1.50 [-6.96, 9.96]
Melkas et al. (2011)	72.5	7.6	50	70.4	7.3	213	4.2%	2.10 [-0.22, 4.42]
Oldenbeuving et al. (2011)	78	8.7	62	71	12.4	465	4.0%	7.00 [4.56, 9.44]
Kara et al. (2013)	68	1.87	42	61.2	1.29	108	5.8%	6.80 [6.18, 7.42]
Turco et al. (2013)	81.9	6.1	58	81.6	6.6	118	4.6%	0.30 [-1.67, 2.27]
Miu & Yeung (2013)	78.8	8.9	86	70.7	9.9	228	4.2%	8.10 [5.82, 10.38]
Naidech et al. (2013)	63.9	12.1	31	61.7	14.6	67	1.7%	2.20 [-3.31, 7.71]
Kozak et al. (2016)	72.91	5.61	11	64.63	13.18	49	2.0%	8.28 [3.32, 13.24]
Alvarez-Perez et al. (2017)	79.7	9	118	74.64	11.13	954	4.8%	5.06 [3.29, 6.83]
Hosoya et al. (2018)	75	1.3	97	69.3	1	172	5.9%	5.70 [5.40, 6.00]
Pasinska et al. (2018)	76.98	10.73	203	69.81	10.42	547	4.8%	7.17 [5.46, 8.88]
Qu et al. (2018)	69.2	16.2	38	60	14.1	223	1.8%	9.20 [3.73, 14.67]
Kotfis et al. (2019)	75.95	13.49	121	70.82	12.15	639	3.9%	5.13 [2.55, 7.71]
Lim et al. (2019)	70.6	9.3	38	69.4	13.4	538	3.3%	1.20 [-1.97, 4.37]
Aizen et al. (2019)	81.5	8.6	30	79.7	7.8	80	3.0%	1.80 [-1.72, 5.32]
Zaitun et al. (2019)	68.6	9.6	15	58.8	11.2	59	1.7%	9.80 [4.16, 15.44]
Ng et al. (2019)	71	12.4	36	62.5	13.6	244	2.3%	8.50 [4.10, 12.90]
Sauvigny et al. (2019)	56.6	13.4	78	53.8	13.4	134	2.8%	2.80 [-0.94, 6.54]
Shaw et al. (2019)	78.64	12.77	105	65.35	11.67	290	3.7%	13.29 [10.50, 16.08]
Rollo et al. (2021)	73	12.8	36	71.3	12.5	84	2.0%	1.70 [-3.26, 6.66]
Reimann et al. (2021)	56	14	65	56	14	162	2.6%	0.00 [-4.03, 4.03]
Fialho Silva et al. (2021)	67.6	11.9	41	59.9	13.1	156	2.5%	7.70 [3.52, 11.88]
Stokholm et al. (2021)	76.9	4.6	8	69	9.9	58	2.6%	7.90 [3.82, 11.98]
Fleischmann et al. (2021)	75.8	13.8	55	71.6	10.5	86	2.4%	4.20 [-0.07, 8.47]
Mengel et al. (2021)	80	10	152	72	15	421	4.4%	8.00 [5.86, 10.14]
Reznik et al. (2022)	72.1	15.5	348	68.3	15.2	242	4.0%	3.80 [1.29, 6.31]
Pendlebury et al. (2022)	84.9	8.9	67	76.7	11.2	127	3.6%	8.20 [5.31, 11.09]
**Total (95% CI)**			**2106**			**6769**	**100.0%**	**5.27 [4.41, 6.14]**

Heterogeneity: Tau² = 3.24; Chi² = 146.69, df = 29 (P < 0.00001); I² = 80%
Test for overall effect: Z = 11.98 (P < 0.00001)

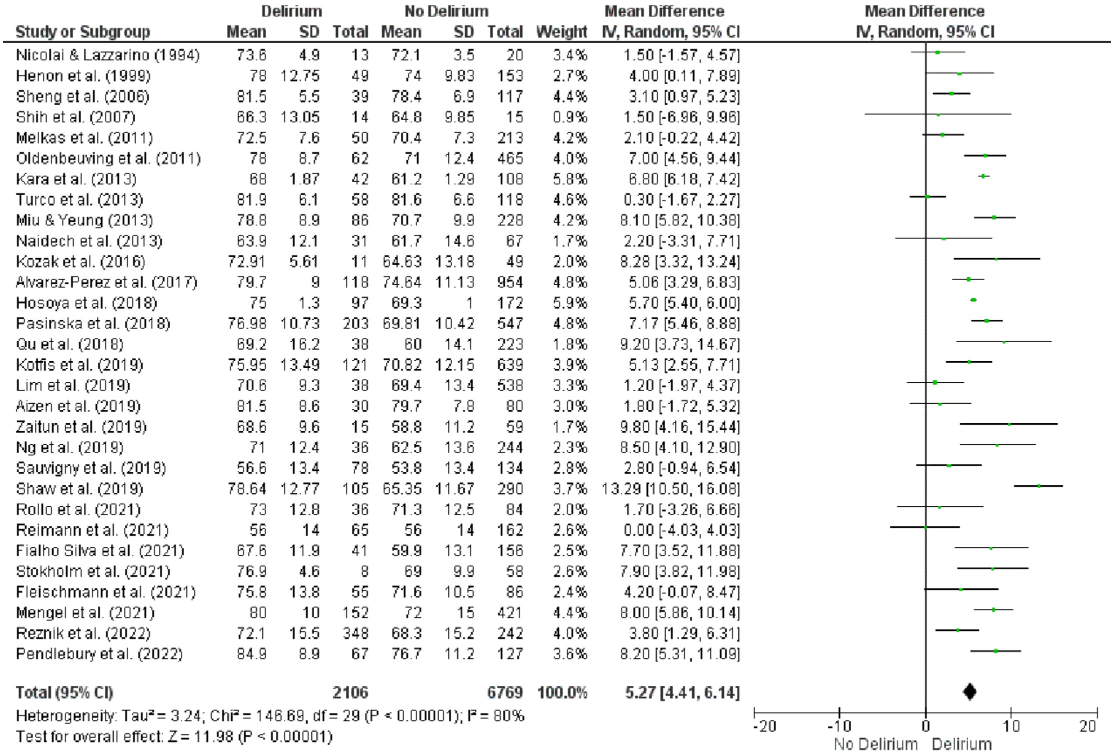

Figure S1a. The Forest Plot presenting the results from meta-analysis of age and PSD

Study or Subgroup	Delirium Events	Delirium Total	No Delirium Events	No Delirium Total	Weight	Odds Ratio IV, Random, 95% CI
Gustafson et al. (1991)	44	69	46	76	2.4%	1.15 [0.59, 2.25]
Henon et al. (1999)	26	49	71	153	2.6%	1.31 [0.69, 2.49]
Caerio et al. (2004)	17	29	113	189	1.9%	0.95 [0.43, 2.11]
Sheng et al. (2006)	17	39	66	117	2.1%	0.60 [0.29, 1.24]
Shih et al. (2007)	10	14	15	15	0.2%	0.08 [0.00, 1.55]
McManus et al. (2009)	15	23	36	59	1.2%	1.20 [0.44, 3.27]
Dahl et al. (2010)	7	18	95	160	1.2%	0.44 [0.16, 1.18]
Oldenbeuving et al. (2011)	38	62	250	465	3.3%	1.36 [0.79, 2.34]
Melkas et al. (2011)	26	50	109	213	2.8%	1.03 [0.56, 1.91]
Kostalova et al. (2012)	24	43	30	57	1.9%	1.14 [0.51, 2.52]
Kara et al. (2013)	30	42	75	108	1.9%	1.10 [0.50, 2.41]
Turco et al. (2013)	26	58	32	118	2.5%	2.18 [1.13, 4.21]
Kozak et al. (2016)	5	11	24	49	0.8%	0.87 [0.23, 3.22]
Ojagbemi et al. (2017)	21	33	31	66	1.6%	1.98 [0.84, 4.66]
Alvarez-Perez et al. (2017)	71	118	506	954	5.1%	1.34 [0.91, 1.98]
Hosoya et al. (2018)	48	97	98	172	3.7%	0.74 [0.45, 1.22]
Qu et al. (2018)	28	38	156	223	1.9%	1.20 [0.55, 2.61]
Kotfis et al. (2018)	78	172	445	829	6.0%	0.72 [0.51, 1.00]
Lim et al. (2019)	28	38	339	538	2.1%	1.64 [0.78, 3.46]
Pasinska et al. (2019)	84	203	268	547	6.1%	0.73 [0.53, 1.02]
Aizen et al. (2019)	16	30	37	80	1.7%	1.33 [0.57, 3.08]
Zaitun et al. (2019)	7	15	33	59	1.0%	0.69 [0.22, 2.15]
Ng et al. (2019)	21	36	165	244	2.2%	0.67 [0.33, 1.37]
Sauvigny et al. (2019)	35	78	43	134	3.1%	1.72 [0.97, 3.06]
Shaw et al. (2019)	48	105	173	290	4.3%	0.57 [0.36, 0.89]
Nakamizo et al. (2021)	27	42	190	345	2.5%	1.47 [0.75, 2.86]
Rollo et al. (2021)	21	36	51	84	1.9%	0.91 [0.41, 2.00]
Reimann et al. (2021)	26	65	64	162	3.0%	1.02 [0.57, 1.84]
Guldof et al. (2021)	101	201	176	313	5.6%	0.79 [0.55, 1.12]
Fialho Silva et al. (2021)	38	71	83	156	3.2%	1.01 [0.58, 1.78]
Marrama et al. (2021)	39	74	95	174	3.3%	0.93 [0.54, 1.60]
Stokholm et al. (2021)	6	8	36	58	0.5%	1.83 [0.34, 9.90]
Fleischmann et al. (2021)	33	55	47	86	2.4%	1.24 [0.63, 2.47]
Mengel et al. (2021)	84	152	225	421	5.3%	1.08 [0.74, 1.56]
Reznik et al. (2022)	181	348	127	242	6.0%	0.98 [0.71, 1.36]
Pendlebury et al. (2022)	29	67	71	127	2.9%	0.60 [0.33, 1.09]
**Total (95% CI)**		**2589**		**8083**	**100.0%**	**0.98 [0.87, 1.11]**
Total events	1355		4421			

Heterogeneity: $Tau^2 = 0.03$; $Chi^2 = 48.29$, df = 35 (P = 0.07); $I^2 = 28\%$

Test for overall effect: Z = 0.27 (P = 0.79)

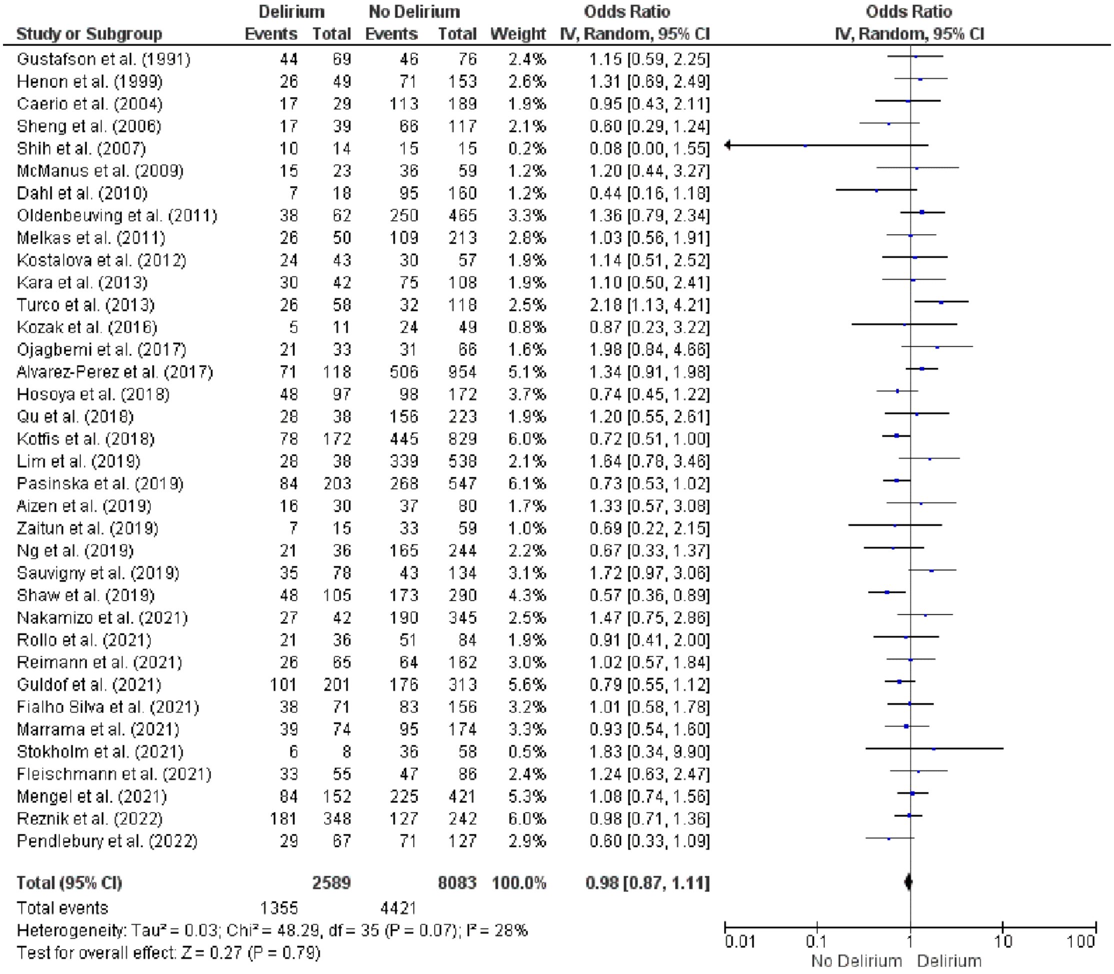

Figure S1b. The Forest Plot presenting the results from meta-analysis of sex and PSD

Study or Subgroup	Delirium Events	Delirium Total	No Delirium Events	No Delirium Total	Weight	Odds Ratio IV, Fixed, 95% CI
Gustafson et al. (1991)	26	69	36	76	3.7%	0.67 [0.35, 1.30]
Nicolai & Lazzarino (1994)	5	13	7	20	0.8%	1.16 [0.27, 4.93]
Henon et al. (1999)	32	49	79	153	3.6%	1.76 [0.90, 3.44]
Sheng et al. (2006)	31	39	80	117	2.1%	1.79 [0.75, 4.28]
Shih et al. (2007)	9	14	13	15	0.5%	0.28 [0.04, 1.76]
Melkas et al. (2011)	20	50	101	213	4.1%	0.74 [0.40, 1.38]
Kara et al. (2013)	27	42	75	108	2.9%	0.79 [0.37, 1.68]
Kozak et al. (2016)	9	11	36	49	0.6%	1.63 [0.31, 8.53]
Ojagbemi et al. (2017)	27	31	47	64	1.2%	2.44 [0.74, 8.01]
Alvarez-Perez et al. (2017)	93	118	771	954	7.3%	0.88 [0.55, 1.41]
Qu et al. (2018)	27	38	148	223	2.9%	1.24 [0.59, 2.64]
Kotfis et al. (2018)	148	172	721	829	7.2%	0.92 [0.57, 1.49]
Hosoya et al. (2018)	45	97	90	172	6.5%	0.79 [0.48, 1.30]
Lim et al. (2019)	29	38	353	538	2.7%	1.69 [0.78, 3.64]
Pasinska et al. (2019)	145	203	388	546	12.8%	1.02 [0.71, 1.45]
Zaitun et al. (2019)	12	15	41	59	0.9%	1.76 [0.44, 6.99]
Ng et al. (2019)	28	36	184	244	2.3%	1.14 [0.49, 2.64]
Sauvigny et al. (2019)	36	78	53	134	5.1%	1.31 [0.75, 2.30]
Rollo et al. (2021)	26	36	60	84	2.1%	1.04 [0.44, 2.48]
Guldof et al. (2021)	156	201	219	313	9.6%	1.49 [0.99, 2.24]
Marrama et al. (2021)	51	74	121	174	4.7%	0.97 [0.54, 1.75]
Reznik et al. (2022)	257	348	164	242	12.5%	1.34 [0.94, 1.93]
Pendlebury et al. (2022)	49	67	78	127	3.9%	1.71 [0.89, 3.27]
**Total (95% CI)**		**1839**		**5454**	**100.0%**	**1.12 [0.99, 1.28]**
Total events	1288		3865			

Heterogeneity: Chi² = 22.10, df = 22 (P = 0.45); I² = 0%
Test for overall effect: Z = 1.80 (P = 0.07)

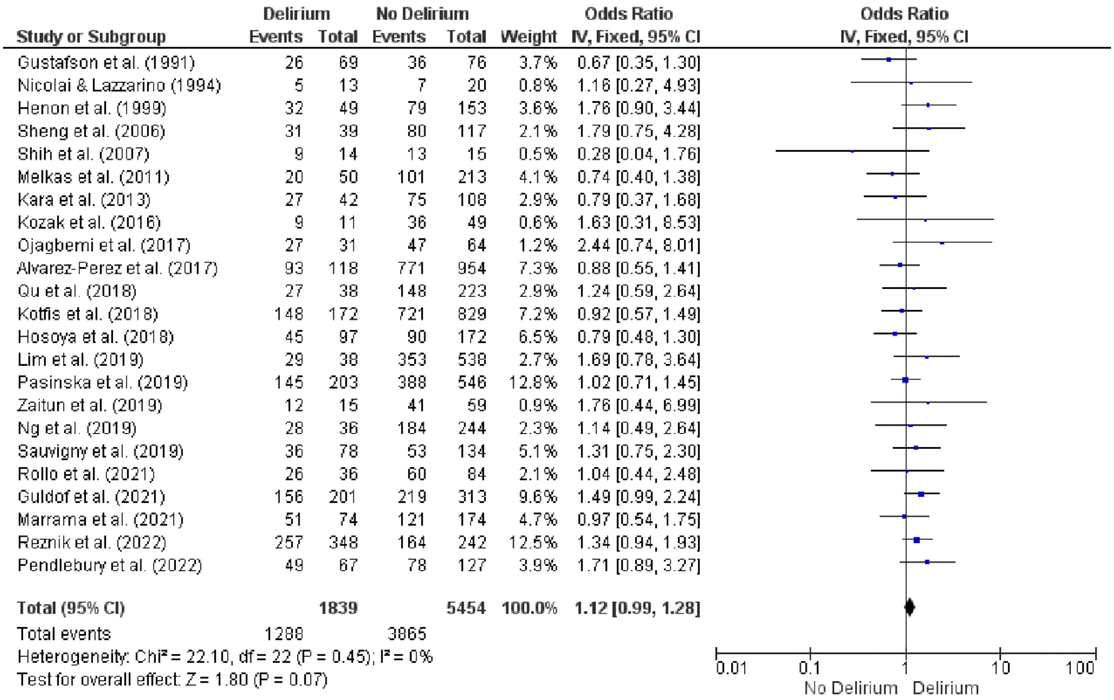

Figure S1c. The Forest Plot presenting the results from meta-analysis of hypertension and PSD

Study or Subgroup	Delirium Events	Delirium Total	No Delirium Events	No Delirium Total	Weight	Odds Ratio IV, Fixed, 95% CI
Henon et al. (1999)	13	49	27	153	7.7%	1.69 [0.79, 3.60]
Caerio et al. (2004)	7	29	76	189	5.5%	0.47 [0.19, 1.16]
Sheng et al. (2006)	20	39	63	117	8.4%	0.90 [0.44, 1.86]
Oldenbeuving et al. (2011)	18	62	151	465	13.1%	0.85 [0.48, 1.52]
Kara et al. (2013)	8	39	12	104	4.6%	1.98 [0.74, 5.29]
Ojagbemi et al. (2017)	14	32	29	66	6.1%	0.99 [0.42, 2.32]
Alvarez-Perez et al. (2017)	32	118	272	954	24.0%	0.93 [0.61, 1.43]
Lim et al. (2019)	5	38	30	538	4.3%	2.57 [0.93, 7.05]
Ng et al. (2019)	1	36	1	244	0.6%	6.94 [0.42, 113.53]
Sauvigny et al. (2019)	6	78	7	134	3.5%	1.51 [0.49, 4.67]
Reznik et al. (2020)	2	34	2	26	1.1%	0.75 [0.10, 5.71]
Reimann et al. (2021)	4	65	5	162	2.4%	2.06 [0.54, 7.92]
Nakamizo et al. (2021)	8	42	37	345	6.2%	1.96 [0.84, 4.55]
Marrama et al. (2021)	26	73	41	171	12.5%	1.75 [0.97, 3.18]
**Total (95% CI)**		**734**		**3668**	**100.0%**	**1.19 [0.97, 1.47]**
Total events	164		753			

Heterogeneity: Chi² = 16.87, df = 13 (P = 0.21); I² = 23%
Test for overall effect: Z = 1.65 (P = 0.10)

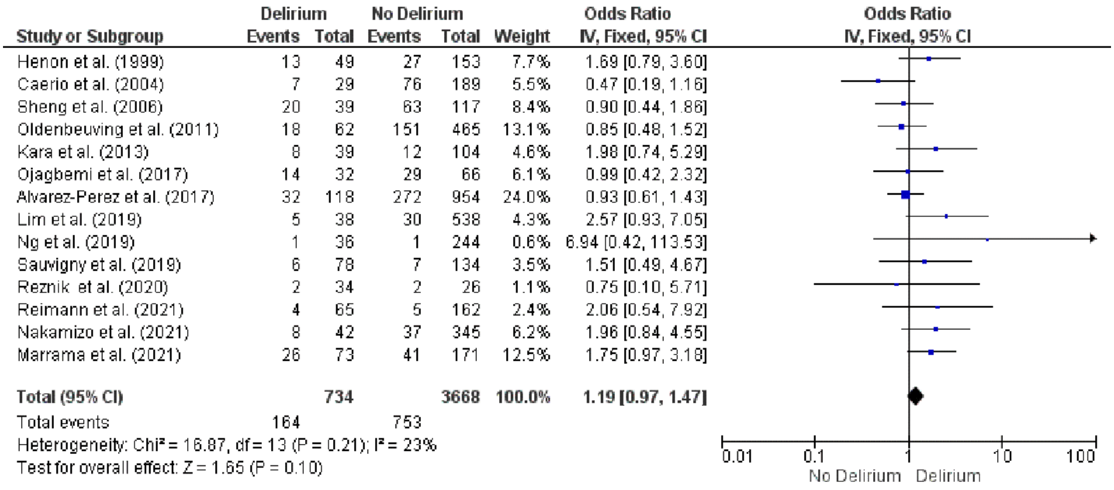

Figure S1d. The Forest Plot presenting the results from meta-analysis of alcohol and PSD

Study or Subgroup	Delirium Events	Delirium Total	No Delirium Events	No Delirium Total	Weight	Odds Ratio IV, Random, 95% CI
Nicolai & Lazzarino (1994)	5	13	9	20	2.7%	0.76 [0.18, 3.17]
Henon et al. (1999)	4	49	32	153	4.2%	0.34 [0.11, 1.00]
Sheng et al. (2006)	15	39	57	117	7.4%	0.66 [0.31, 1.38]
Kara et al. (2013)	12	33	24	92	6.2%	1.62 [0.69, 3.78]
Kozak et al. (2016)	3	11	11	49	2.5%	1.30 [0.29, 5.73]
Ojagbemi et al. (2017)	6	33	9	66	4.0%	1.41 [0.45, 4.36]
Alvarez-Perez et al. (2017)	32	118	256	954	13.0%	1.01 [0.66, 1.56]
Kotfis et al. (2018)	74	172	343	829	15.3%	1.07 [0.77, 1.49]
Lim et al. (2019)	15	38	129	538	8.3%	2.07 [1.05, 4.08]
Pasinska et al. (2019)	24	79	252	531	11.3%	0.48 [0.29, 0.80]
Zaitun et al. (2019)	4	15	23	59	3.4%	0.57 [0.16, 2.00]
Rollo et al. (2021)	5	36	14	84	4.1%	0.81 [0.27, 2.44]
Marrama et al. (2021)	16	73	28	171	8.2%	1.43 [0.72, 2.85]
Pendlebury et al. (2022)	25	67	54	127	9.4%	0.80 [0.44, 1.48]
**Total (95% CI)**		**776**		**3790**	**100.0%**	**0.94 [0.73, 1.21]**
Total events	240		1241			

Heterogeneity: Tau² = 0.08; Chi² = 21.43, df = 13 (P = 0.06); I² = 39%
Test for overall effect: Z = 0.49 (P = 0.62)

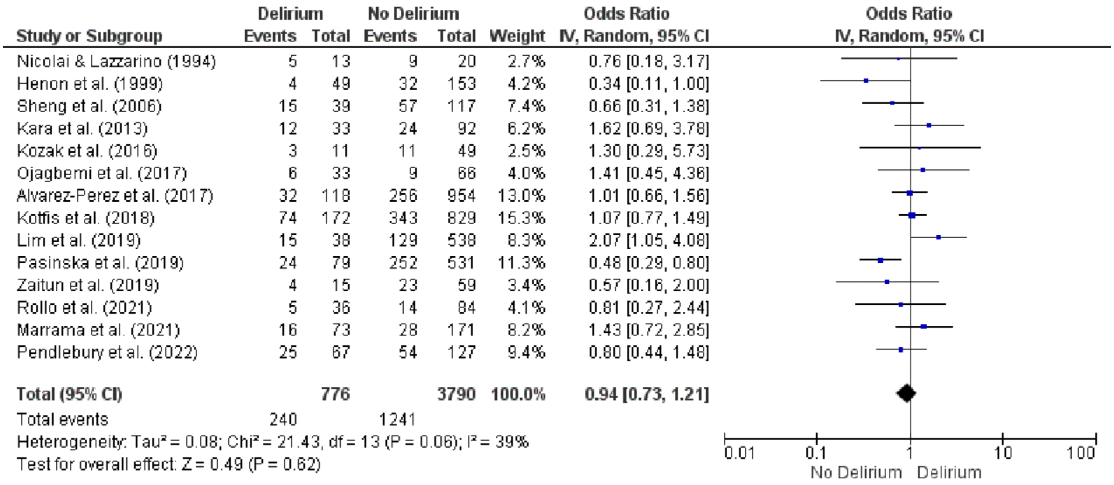

Figure S1e. The Forest Plot presenting the results from meta-analysis of smoking and PSD

Study or Subgroup	Delirium Events	Delirium Total	No Delirium Events	No Delirium Total	Weight	Odds Ratio IV, Fixed, 95% CI
Gustafson et al. (1991)	12	69	12	76	2.4%	1.12 [0.47, 2.70]
Nicolai & Lazzarino (1994)	4	13	4	20	0.7%	1.78 [0.36, 8.88]
Henon et al. (1999)	9	49	15	153	2.2%	2.07 [0.84, 5.08]
Caerio et al. (2004)	6	29	35	189	1.9%	1.15 [0.43, 3.03]
Sheng et al. (2006)	9	39	23	117	2.4%	1.23 [0.51, 2.94]
Shih et al. (2007)	7	14	0	15	0.2%	31.00 [1.55, 618.01]
Dahl et al. (2010)	1	18	11	160	0.4%	0.80 [0.10, 6.56]
Melkas et al. (2011)	10	50	53	213	3.1%	0.75 [0.35, 1.61]
Kara et al. (2013)	14	42	43	106	3.2%	0.73 [0.35, 1.55]
Kozak et al. (2016)	5	11	18	49	1.0%	1.44 [0.38, 5.38]
Ojagbemi et al. (2017)	8	32	18	66	1.9%	0.89 [0.34, 2.34]
Alvarez-Perez et al. (2017)	22	118	225	954	7.6%	0.74 [0.46, 1.21]
Qu et al. (2018)	5	38	53	223	1.8%	0.49 [0.18, 1.31]
Kotfis et al. (2018)	39	172	166	829	11.6%	1.17 [0.79, 1.74]
Hosoya et al. (2018)	23	97	41	172	5.3%	0.99 [0.55, 1.78]
Lim et al. (2019)	18	38	192	538	4.1%	1.62 [0.84, 3.14]
Pasinska et al. (2019)	71	201	132	546	14.8%	1.71 [1.21, 2.43]
Zaitun et al. (2019)	5	15	25	59	1.3%	0.68 [0.21, 2.24]
Ng et al. (2019)	12	36	108	244	3.3%	0.63 [0.30, 1.32]
Sauvigny et al. (2019)	2	78	4	134	0.6%	0.86 [0.15, 4.78]
Rollo et al. (2021)	5	36	23	84	1.6%	0.43 [0.15, 1.23]
Guldof et al. (2021)	49	201	56	313	9.7%	1.48 [0.96, 2.28]
Marrama et al. (2021)	7	74	27	174	2.3%	0.57 [0.24, 1.37]
Stokholm et al. (2021)	1	8	10	58	0.4%	0.69 [0.08, 6.21]
Reznik et al. (2022)	83	348	57	242	12.2%	1.02 [0.69, 1.50]
Pendlebury et al. (2022)	17	67	28	127	3.8%	1.20 [0.60, 2.40]
**Total (95% CI)**		**1893**		**5861**	**100.0%**	**1.10 [0.96, 1.26]**
Total events	444		1379			

Heterogeneity: Chi² = 32.76, df = 25 (P = 0.14); I² = 24%
Test for overall effect: Z = 1.43 (P = 0.15)

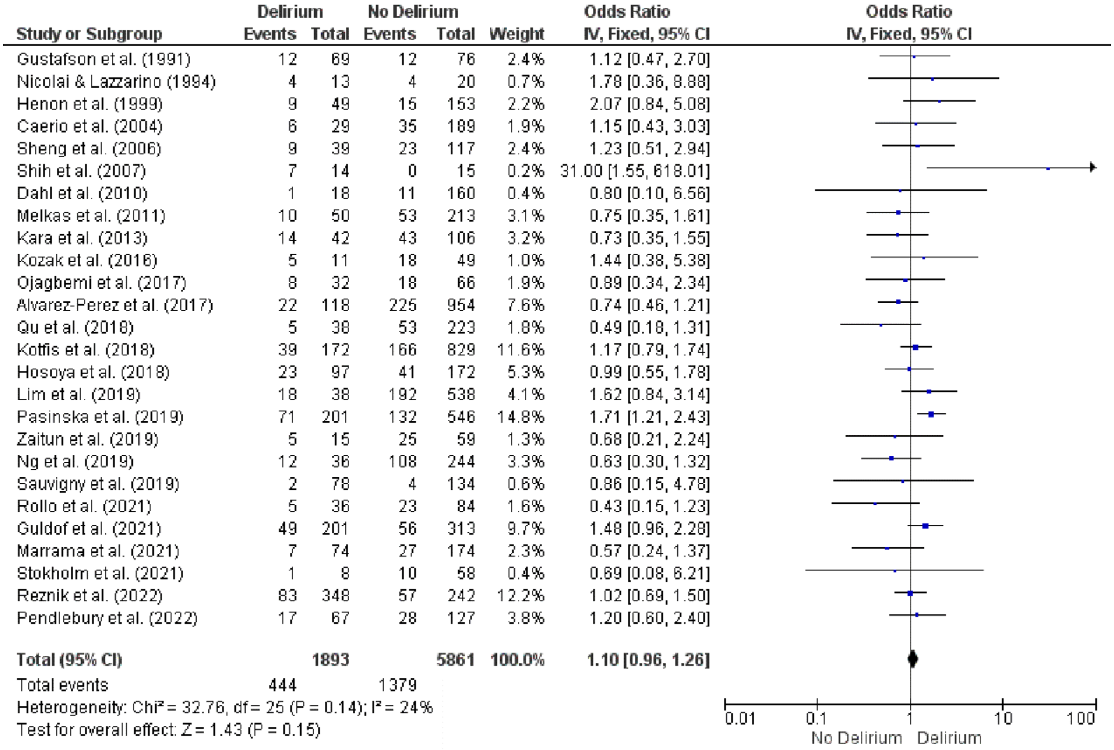

Figure S1f. The Forest Plot presenting the results from meta-analysis of DM and PSD

Study or Subgroup	Delirium Events	Delirium Total	No Delirium Events	No Delirium Total	Weight	Odds Ratio IV, Random, 95% CI
Henon et al. (1999)	17	49	41	153	7.6%	1.45 [0.73, 2.89]
Sheng et al. (2006)	17	39	32	117	7.1%	2.05 [0.97, 4.35]
McManus et al. (2009)	9	23	12	59	5.1%	2.52 [0.88, 7.20]
Melkas et al. (2011)	12	50	37	213	7.2%	1.50 [0.72, 3.15]
Kozak et al. (2016)	4	11	10	49	3.4%	2.23 [0.54, 9.14]
Alvarez-Perez et al. (2017)	47	118	242	954	10.3%	1.95 [1.31, 2.89]
Kotfis et al. (2018)	83	172	210	829	10.8%	2.75 [1.96, 3.85]
Pasinska et al. (2019)	76	202	104	546	10.7%	2.56 [1.80, 3.66]
Ng et al. (2019)	8	36	31	244	6.2%	1.96 [0.82, 4.69]
Rollo et al. (2021)	15	36	17	84	6.4%	2.82 [1.20, 6.59]
Marrama et al. (2021)	9	74	29	174	6.7%	0.69 [0.31, 1.55]
Reznik et al. (2022)	73	348	66	242	10.4%	0.71 [0.48, 1.04]
Pendlebury et al. (2022)	25	67	32	127	8.1%	1.77 [0.93, 3.34]
**Total (95% CI)**		**1225**		**3791**	**100.0%**	**1.74 [1.28, 2.36]**
Total events	395		863			

Heterogeneity: Tau² = 0.20; Chi² = 40.55, df = 12 (P < 0.0001); I² = 70%
Test for overall effect: Z = 3.55 (P = 0.0004)

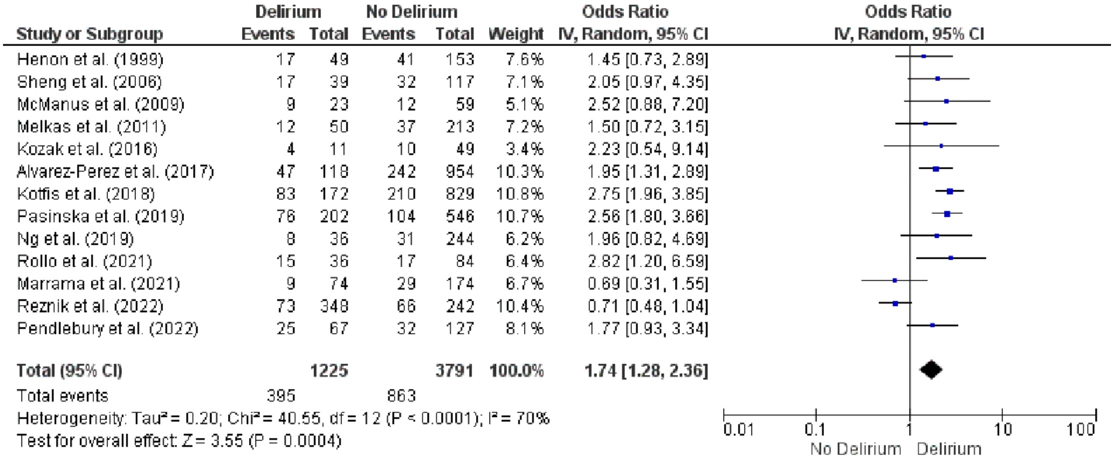

Figure S1g. The Forest Plot presenting the results from meta-analysis of AF and PSD

Study or Subgroup	Delirium Events	Delirium Total	No Delirium Events	No Delirium Total	Weight	Odds Ratio IV, Fixed, 95% CI
Henon et al. (1999)	7	49	38	153	10.2%	0.50 [0.21, 1.22]
Shih et al. (2007)	6	14	8	15	3.7%	0.66 [0.15, 2.84]
Kara et al. (2013)	16	42	41	108	14.6%	1.01 [0.48, 2.10]
Kozak et al. (2016)	5	11	11	49	4.2%	2.88 [0.74, 11.25]
Ng et al. (2019)	14	36	86	244	15.2%	1.17 [0.57, 2.40]
Sauvigny et al. (2019)	7	78	10	134	7.7%	1.22 [0.45, 3.35]
Marrama et al. (2021)	22	74	53	174	22.3%	0.97 [0.53, 1.75]
Pendlebury et al. (2022)	29	67	56	127	22.1%	0.97 [0.53, 1.76]
**Total (95% CI)**		**371**		**1004**	**100.0%**	**0.99 [0.74, 1.30]**
Total events	106		303			

Heterogeneity: Chi² = 5.30, df = 7 (P = 0.62); I² = 0%
Test for overall effect: Z = 0.11 (P = 0.92)

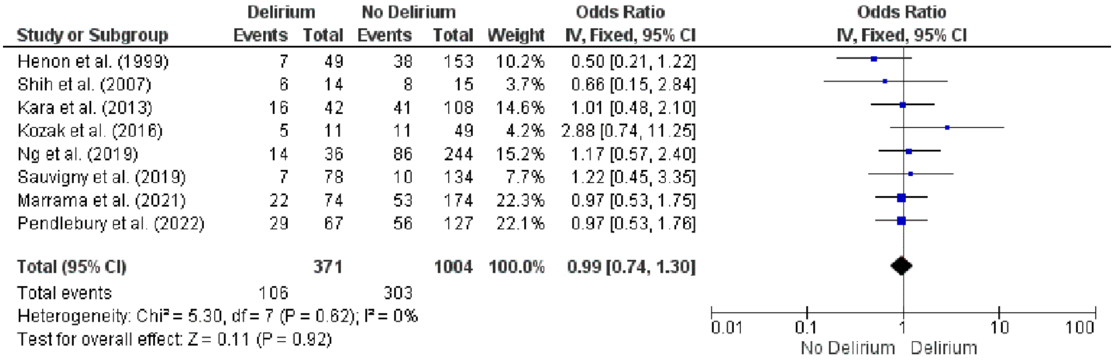

Figure S1h. The Forest Plot presenting the results from meta-analysis of hyperlipidemia and PSD

	Delirium		No Delirium			Odds Ratio
**Study or Subgroup**	**Events**	**Total**	**Events**	**Total**	**Weight**	**IV, Fixed, 95% CI**
Gustafson et al. (1991)	25	69	20	76	3.9%	1.59 [0.78, 3.23]
Henon et al. (1999)	14	49	41	153	3.8%	1.09 [0.53, 2.24]
Caerio et al. (2004)	5	29	35	189	1.8%	0.92 [0.33, 2.57]
Sheng et al. (2006)	14	39	31	117	3.3%	1.55 [0.72, 3.36]
McManus et al. (2009)	8	23	9	59	1.6%	2.96 [0.97, 9.02]
Dahl et al. (2010)	5	18	37	160	1.6%	1.28 [0.43, 3.82]
Kara et al. (2013)	11	42	17	108	2.6%	1.90 [0.80, 4.49]
Ojagbemi et al. (2017)	3	33	6	66	0.9%	1.00 [0.23, 4.28]
Alvarez-Perez et al. (2017)	25	118	197	954	8.9%	1.03 [0.65, 1.65]
Qu et al. (2018)	14	38	32	223	3.4%	3.48 [1.63, 7.43]
Kotfis et al. (2018)	50	172	172	829	14.3%	1.57 [1.08, 2.27]
Aizen et al. (2019)	8	30	20	80	2.1%	1.09 [0.42, 2.83]
Zaitun et al. (2019)	2	15	13	59	0.8%	0.54 [0.11, 2.73]
Ng et al. (2019)	9	36	54	244	3.0%	1.17 [0.52, 2.64]
Shaw et al. (2019)	34	105	83	290	8.4%	1.19 [0.74, 1.93]
Czyzycki et al. (2021)	37	166	98	515	10.7%	1.22 [0.80, 1.87]
Rollo et al. (2021)	6	36	9	84	1.6%	1.67 [0.55, 5.09]
Guldof et al. (2021)	54	201	48	313	10.2%	2.03 [1.31, 3.14]
Marrama et al. (2021)	16	74	28	174	4.2%	1.44 [0.72, 2.85]
Reznik et al. (2022)	86	348	38	242	10.9%	1.76 [1.15, 2.69]
Pendlebury et al. (2022)	9	67	10	127	2.1%	1.82 [0.70, 4.71]
**Total (95% CI)**		**1708**		**5062**	**100.0%**	**1.48 [1.28, 1.70]**
Total events	435		998			

Heterogeneity: Chi² = 17.58, df = 20 (P = 0.61); I² = 0%
Test for overall effect: Z = 5.46 (P < 0.00001)

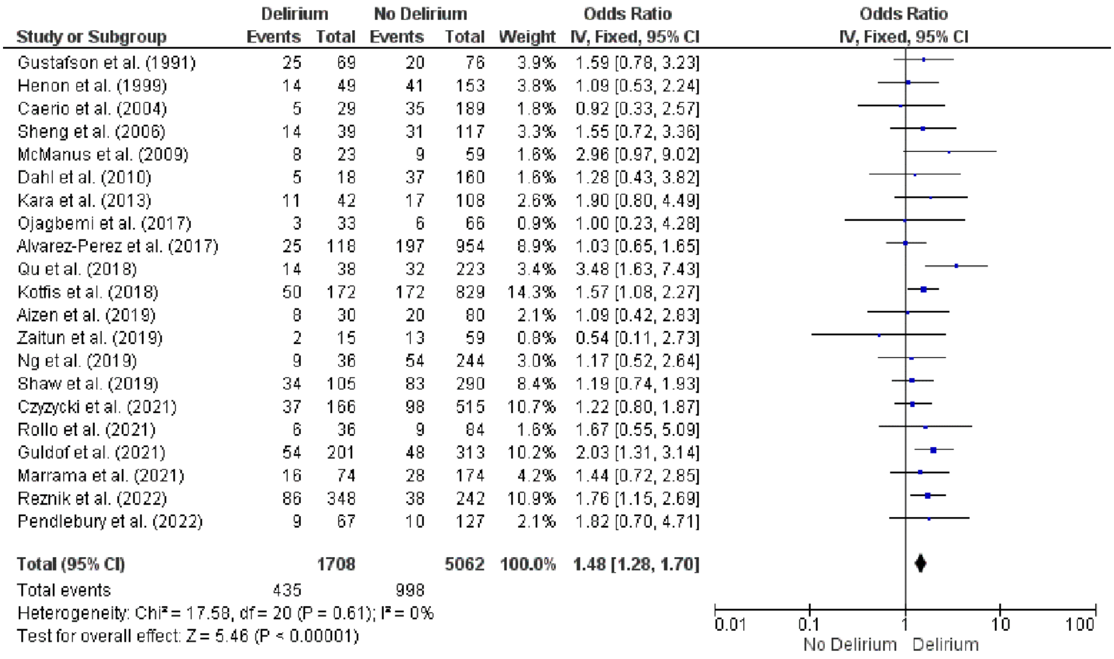

Figure S1i. The Forest Plot presenting the results from meta-analysis of prior stroke and PSD

	Delirium		No Delirium			Odds Ratio
**Study or Subgroup**	**Events**	**Total**	**Events**	**Total**	**Weight**	**IV, Fixed, 95% CI**
Henon et al. (1999)	18	49	42	153	6.5%	1.53 [0.78, 3.03]
Sheng et al. (2006)	12	39	35	117	4.9%	1.04 [0.47, 2.29]
Melkas et al. (2011)	8	50	40	213	4.4%	0.82 [0.36, 1.89]
Kara et al. (2013)	20	42	28	108	5.5%	2.60 [1.24, 5.46]
Kozak et al. (2016)	12	49	6	11	1.6%	0.27 [0.07, 1.05]
Alvarez-Perez et al. (2017)	16	118	114	954	9.6%	1.16 [0.66, 2.03]
Kotfis et al. (2018)	60	172	198	829	24.4%	1.71 [1.20, 2.43]
Ng et al. (2019)	8	36	40	244	4.1%	1.46 [0.62, 3.43]
Pasinska et al. (2019)	33	203	73	547	15.1%	1.26 [0.81, 1.97]
Marrama et al. (2021)	17	74	28	174	6.6%	1.56 [0.79, 3.06]
Reznik et al. (2022)	66	348	25	242	12.4%	2.03 [1.24, 3.33]
Pendlebury et al. (2022)	14	67	16	127	4.9%	1.83 [0.83, 4.03]
**Total (95% CI)**		**1247**		**3719**	**100.0%**	**1.48 [1.25, 1.76]**
Total events	284		645			

Heterogeneity: Chi² = 14.71, df = 11 (P = 0.20); I² = 25%
Test for overall effect: Z = 4.44 (P < 0.00001)

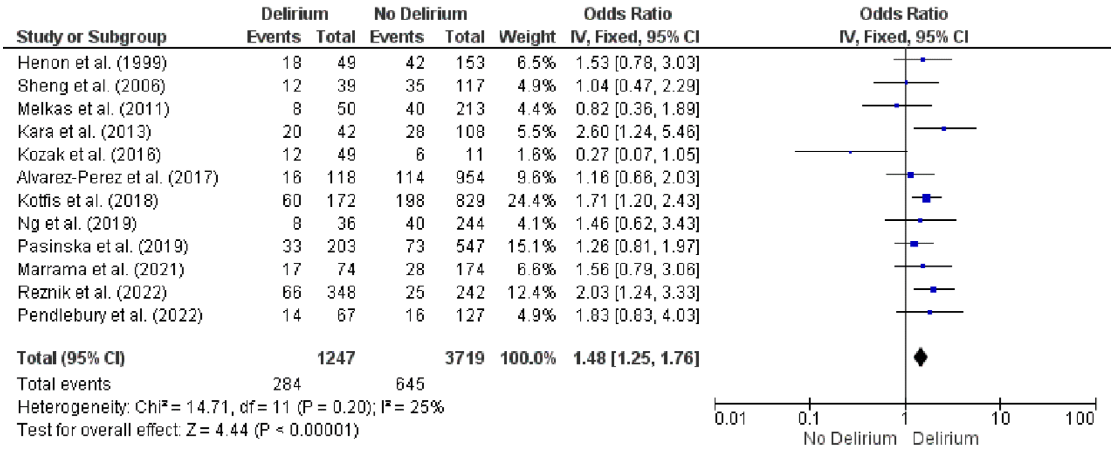

Figure S1g. The Forest Plot presenting the results from meta-analysis of CAD and PSD

Study or Subgroup	Delirium Events	Delirium Total	No Delirium Events	No Delirium Total	Weight	Odds Ratio IV, Fixed, 95% CI
Sheng et al. (2006)	5	39	8	117	7.9%	2.00 [0.61, 6.53]
Melkas et al. (2011)	10	50	41	213	18.5%	1.05 [0.48, 2.27]
Kozak et al. (2016)	3	11	4	49	3.9%	4.22 [0.79, 22.53]
Kotfis et al. (2019)	51	121	156	639	67.6%	2.26 [1.51, 3.38]
Reznik et al. (2020)	4	34	1	26	2.2%	3.33 [0.35, 31.77]
**Total (95% CI)**		**255**		**1044**	**100.0%**	**2.01 [1.44, 2.79]**
Total events	73		210			

Heterogeneity: Chi² = 3.99, df = 4 (P = 0.41); I² = 0%
Test for overall effect: Z = 4.11 (P < 0.0001)

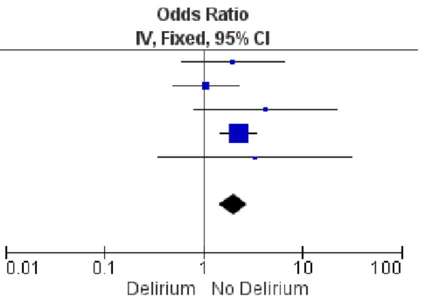

Figure S1k. The Forest Plot presenting the results from meta-analysis of HF and PSD

Study or Subgroup	Delirium Events	Delirium Total	No Delirium Events	No Delirium Total	Weight	Odds Ratio IV, Fixed, 95% CI
Henon et al. (1999)	9	49	13	153	9.4%	2.42 [0.97, 6.08]
Melkas et al. (2011)	5	50	24	213	7.7%	0.88 [0.32, 2.42]
Kotfis et al. (2018)	106	172	348	829	70.1%	2.22 [1.59, 3.11]
Pendlebury et al. (2022)	14	67	16	127	12.8%	1.83 [0.83, 4.03]
**Total (95% CI)**		**338**		**1322**	**100.0%**	**2.03 [1.53, 2.70]**
Total events	134		401			

Heterogeneity: Chi² = 3.11, df = 3 (P = 0.38); I² = 3%
Test for overall effect: Z = 4.93 (P < 0.00001)

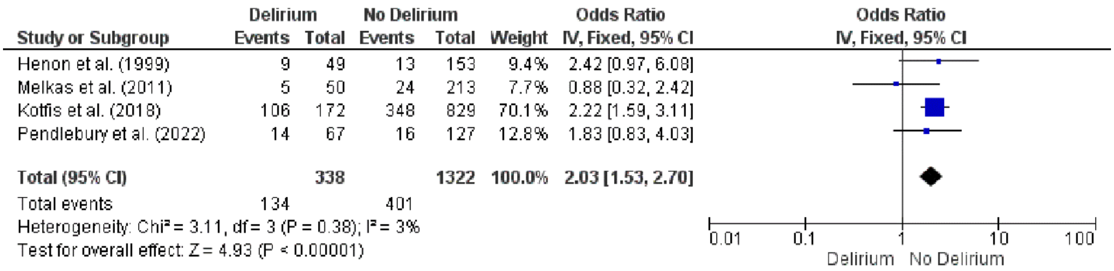

Figure S1l. The Forest Plot presenting the results from meta-analysis of PVD and PSD
