# Supplementary material for: The Role of Vascular Risk Factors in Post-Stroke Delirium: A Systematic Review and Meta-Analysis
Source: J Clin Med. 2022 Oct 1;11(19):5835. doi: 10.3390/jcm11195835 (PMC9571874; doi:10.3390/jcm11195835)
Supplement: Supplementary file 1 [file jcm-11-05835-s001.zip › Figures S2a to S2l.pdf]

Figures S2a to S2l. The Funnel Plots presenting the results from meta-analysis of vascular risk factors and PSD

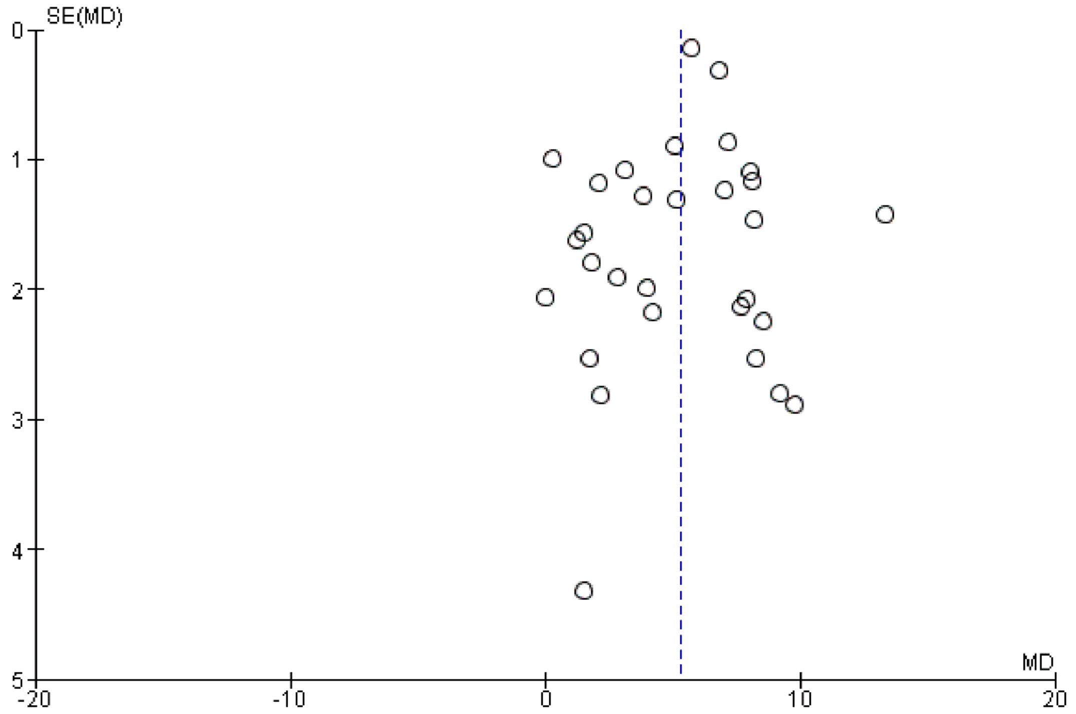

Figure S2a. The Funnel Plot presenting the results from meta-analysis of age and PSD

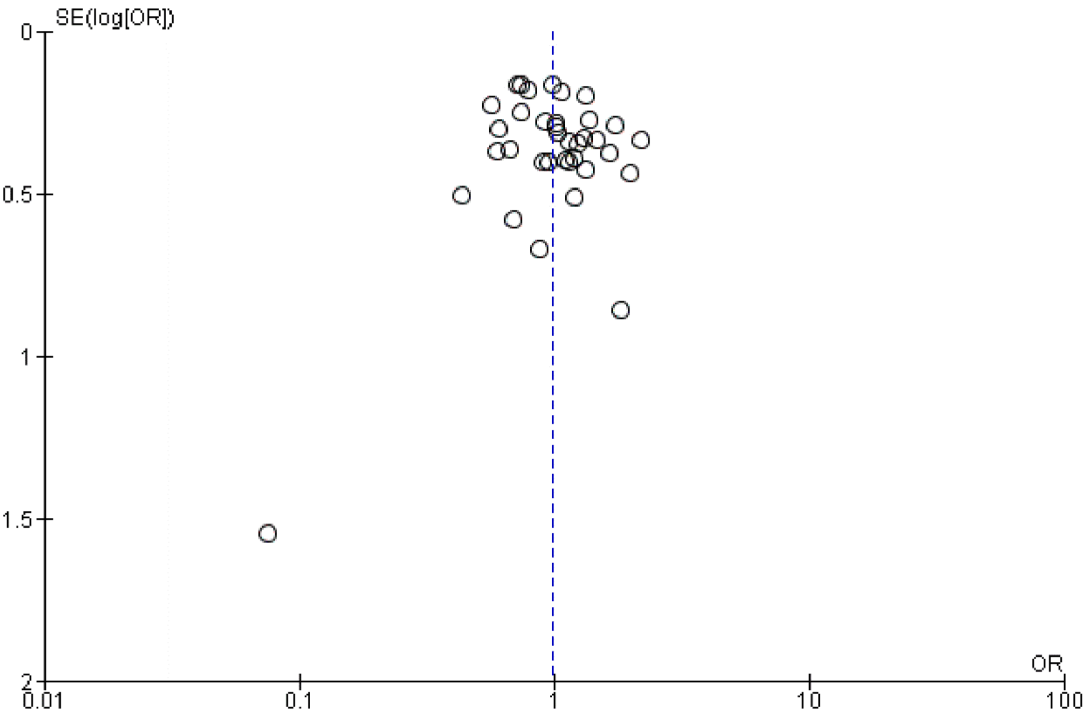

Figure S2b. The Funnel Plot presenting the results from meta-analysis of sex and PSD

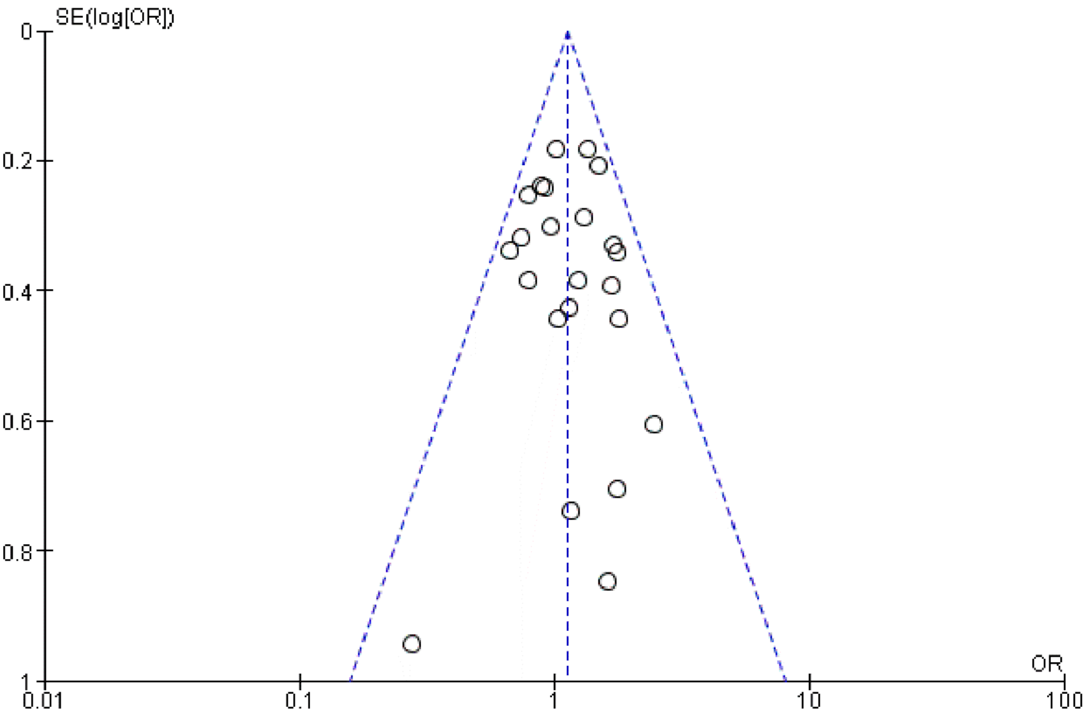

Figure S2c. The Funnel Plot presenting the results from meta-analysis of hypertension and PSD

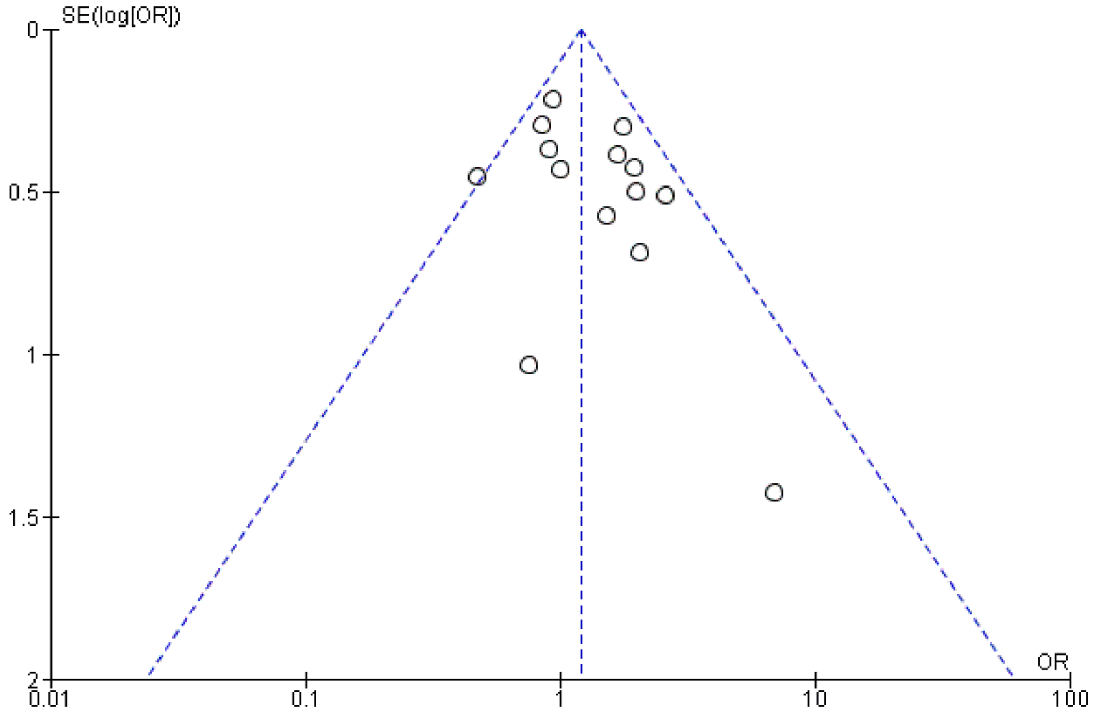

Figure S2d. The Funnel Plot presenting the results from meta-analysis of alcohol and PSD

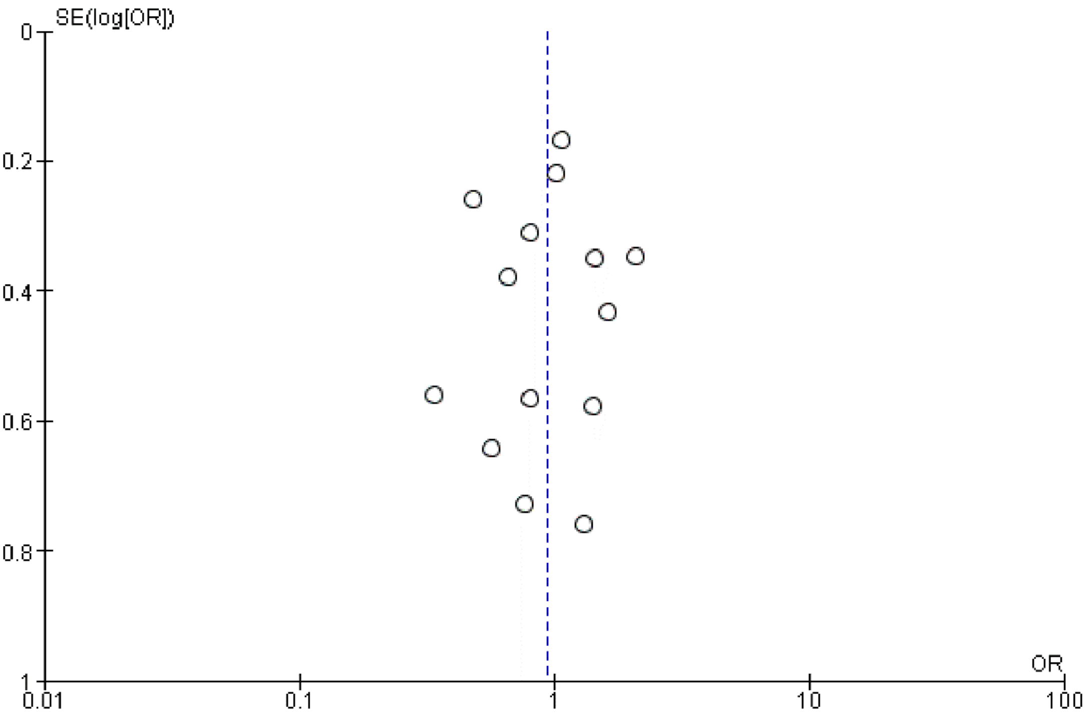

Figure S2e. The Funnel Plot presenting the results from meta-analysis of smoking and PSD

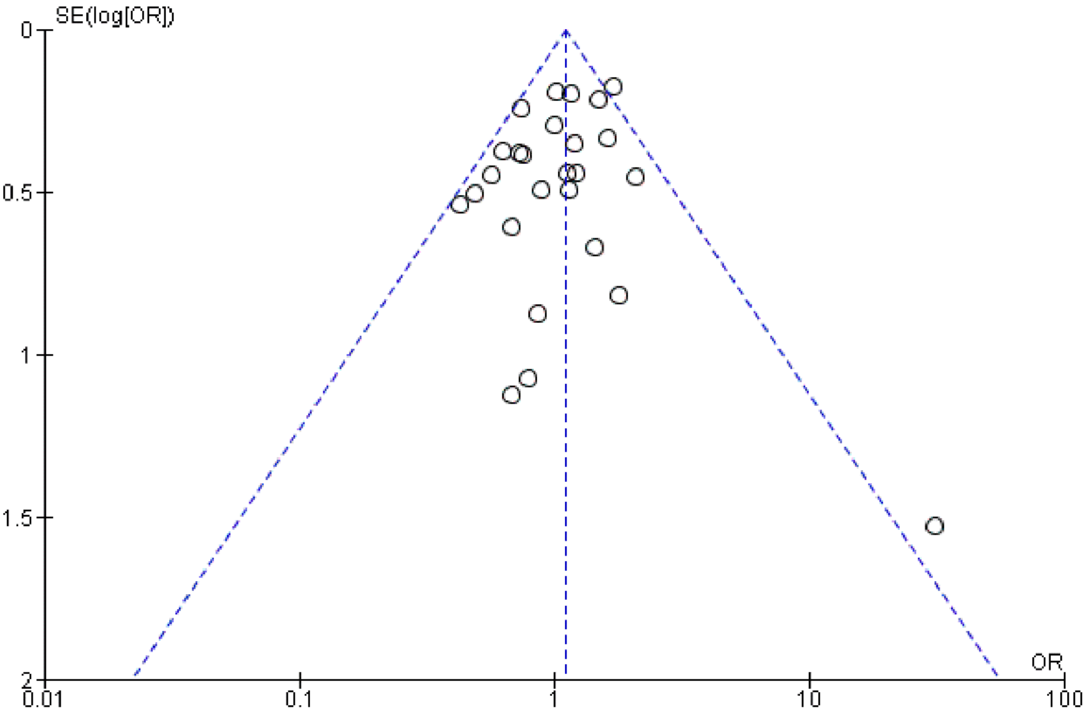

Figure S2f. The Funnel Plot presenting the results from meta-analysis of DM and PSD

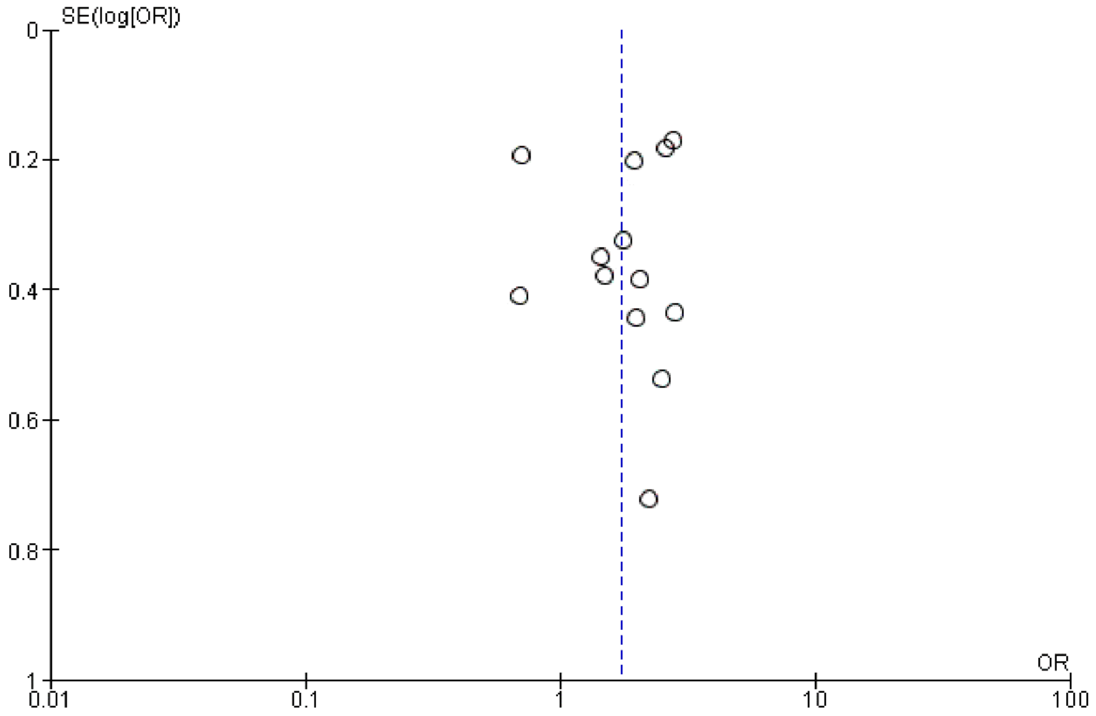

Figure S2g. The Funnel Plot presenting the results from meta-analysis of AF and PSD

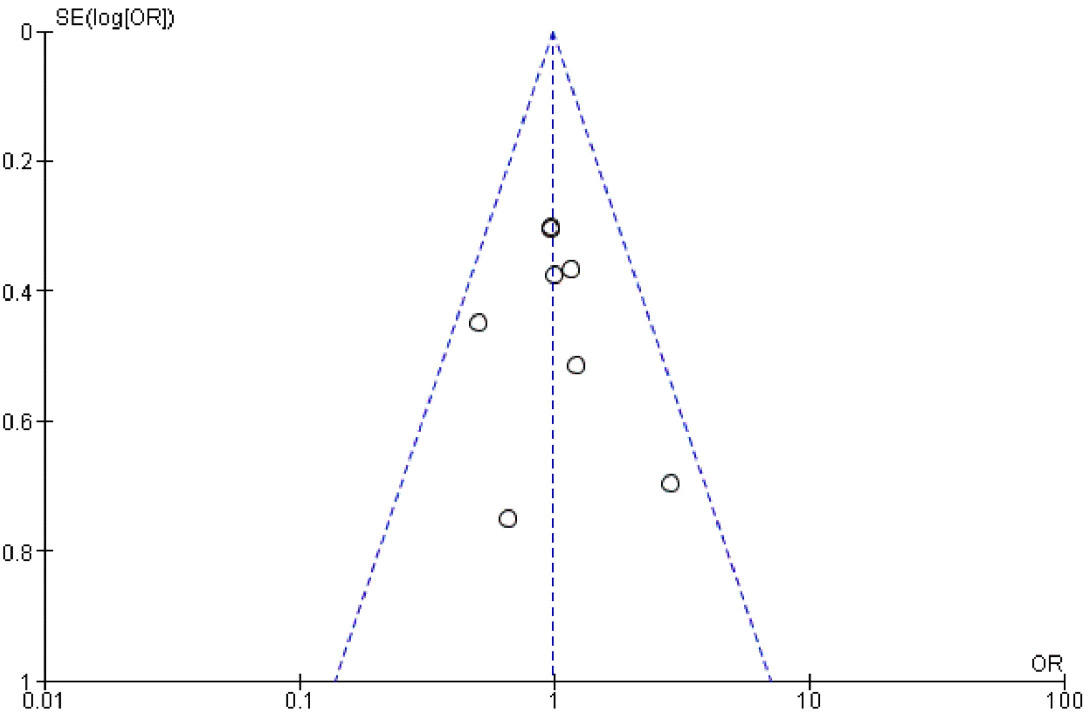

Figure S2h. The Funnel Plot presenting the results from meta-analysis of hyperlipidemia and PSD

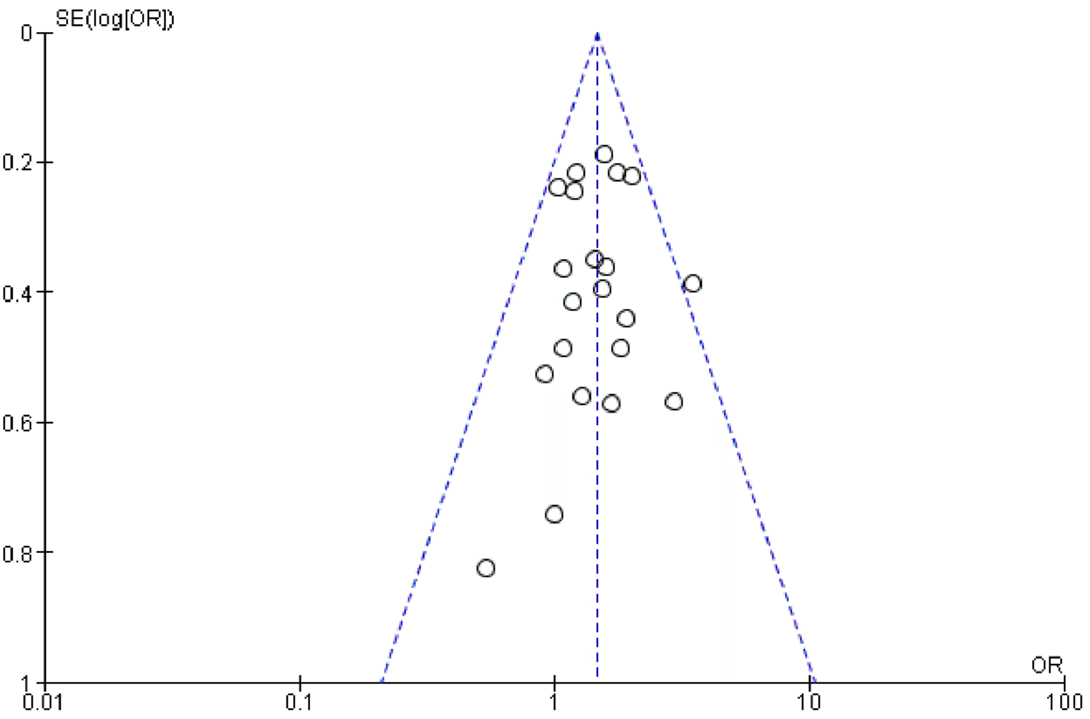

Figure S2i. The Funnel Plot presenting the results from meta-analysis of prior stroke and PSD

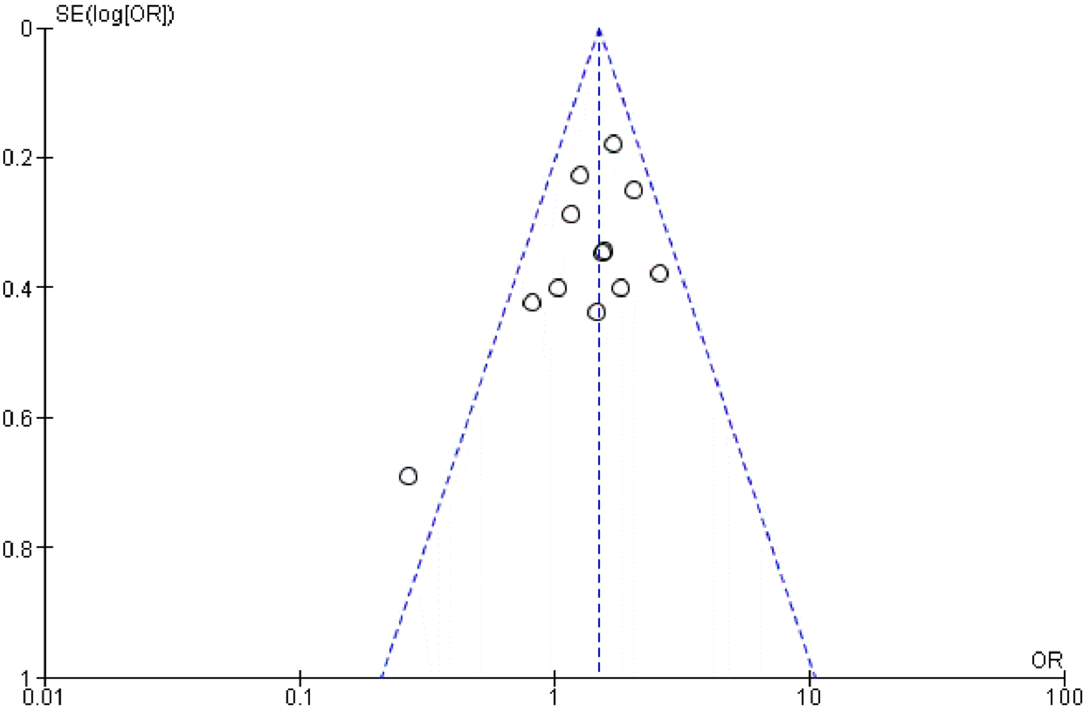

Figure S2j. The Funnel Plot presenting the results from meta-analysis of CAD and PSD

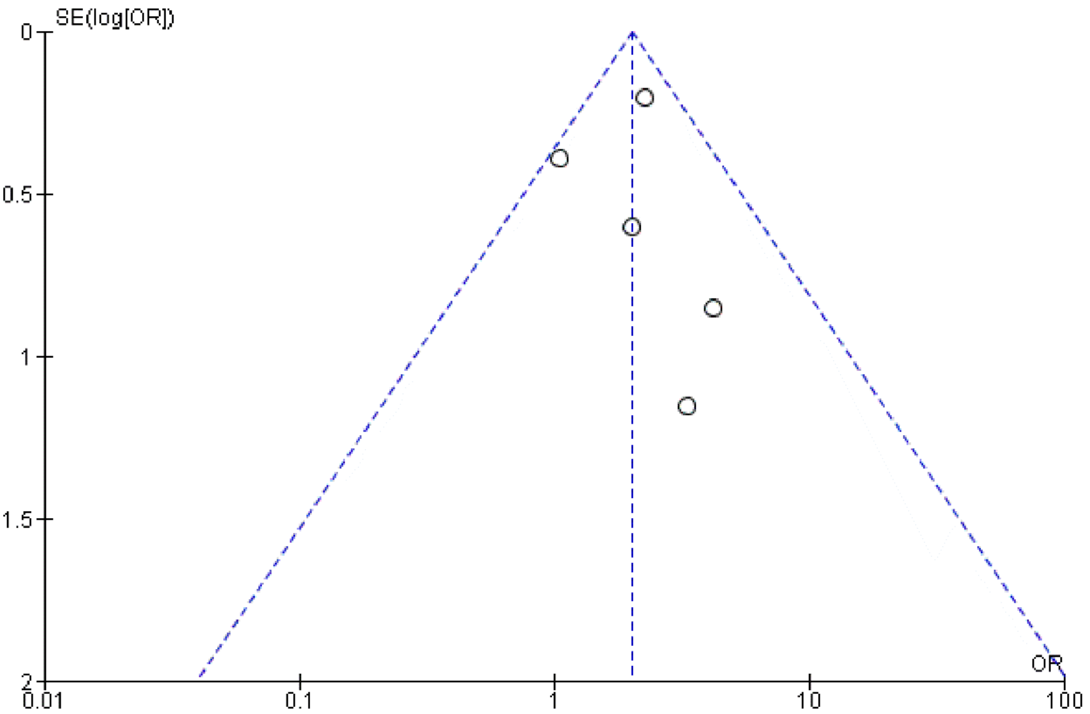

Figure S2k. The Funnel Plot presenting the results from meta-analysis of HF and PSD

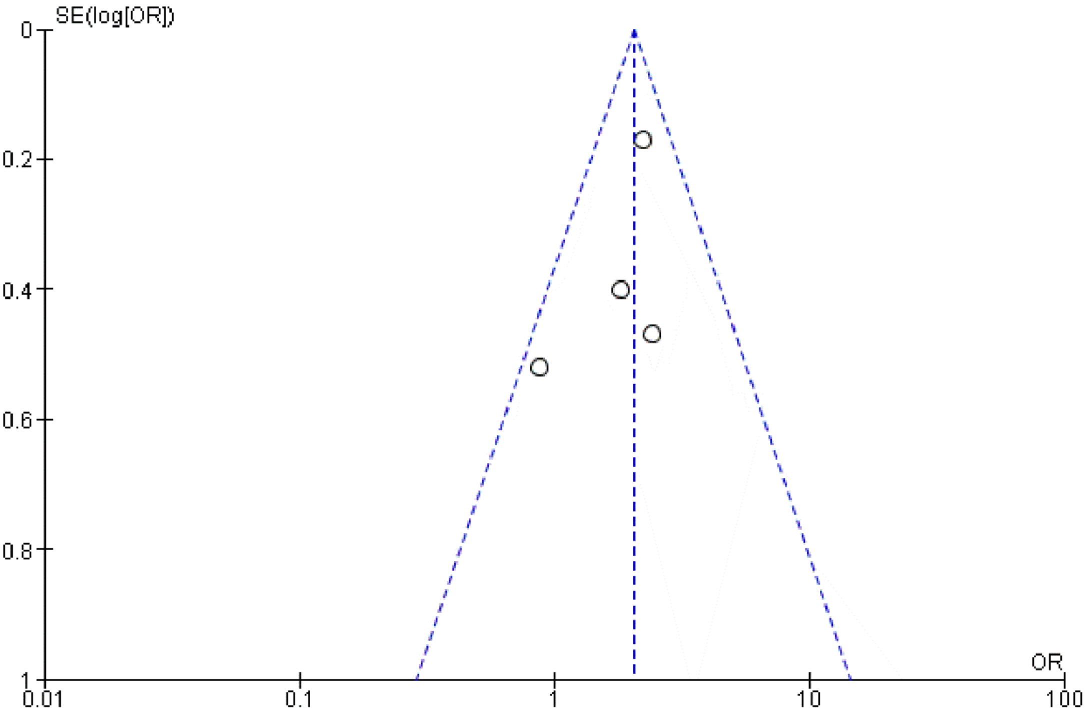

Figure S2l. The Funnel Plot presenting the results from meta-analysis of PVD and PSD
