# Supplementary material for: The Role of Vascular Risk Factors in Post-Stroke Delirium: A Systematic Review and Meta-Analysis
Source: J Clin Med. 2022 Oct 1;11(19):5835. doi: 10.3390/jcm11195835 (PMC9571874; doi:10.3390/jcm11195835)
Supplement: Supplementary file 1 [file jcm-11-05835-s001.zip › Figures S3a to S3f.pdf]

Figures S3a to S3f. The Forest Plots presenting the results from meta-analysis of age, AF, prior stroke, CAD, HF, PVD and PSD in stroke/TIA subgroup

	Delirium			No Delirium				Mean Difference
Study or Subgroup	Mean	SD	Total	Mean	SD	Total	Weight	IV, Random, 95% CI
Nicolai & Lazzarino (1994)	73.6	4.9	13	72.1	3.5	20	9.2%	1.50 [-1.57, 4.57]
Shih et al. (2007)	66.3	13.05	14	64.8	9.85	15	2.4%	1.50 [-6.96, 9.96]
Melkas et al. (2011)	72.5	7.6	50	70.4	7.3	213	11.2%	2.10 [-0.22, 4.42]
Kozak et al. (2016)	72.91	5.61	11	64.63	13.18	49	5.4%	8.28 [3.32, 13.24]
Hosoya et al. (2018)	75	1.3	97	69.3	1	172	16.0%	5.70 [5.40, 6.00]
Qu et al. (2018)	69.2	16.2	38	60	14.1	223	4.8%	9.20 [3.73, 14.67]
Kotfis et al. (2019)	75.95	13.49	121	70.82	12.15	639	10.5%	5.13 [2.55, 7.71]
Lim et al. (2019)	70.6	9.3	38	69.4	13.4	538	8.9%	1.20 [-1.97, 4.37]
Ng et al. (2019)	71	12.4	36	62.5	13.6	244	6.3%	8.50 [4.10, 12.90]
Stokholm et al. (2021)	76.9	4.6	8	69	9.9	58	6.9%	7.90 [3.82, 11.98]
Fleischmann et al. (2021)	75.8	13.8	55	71.6	10.5	86	6.6%	4.20 [-0.07, 8.47]
Mengel et al. (2021)	80	10	152	72	15	421	11.8%	8.00 [5.86, 10.14]
**Total (95% CI)**			**633**			**2678**	**100.0%**	**5.16 [3.73, 6.58]**

Heterogeneity: Tau² = 3.27; Chi² = 35.29, df = 11 (P = 0.0002); I² = 69%
Test for overall effect: Z = 7.11 (P < 0.00001)

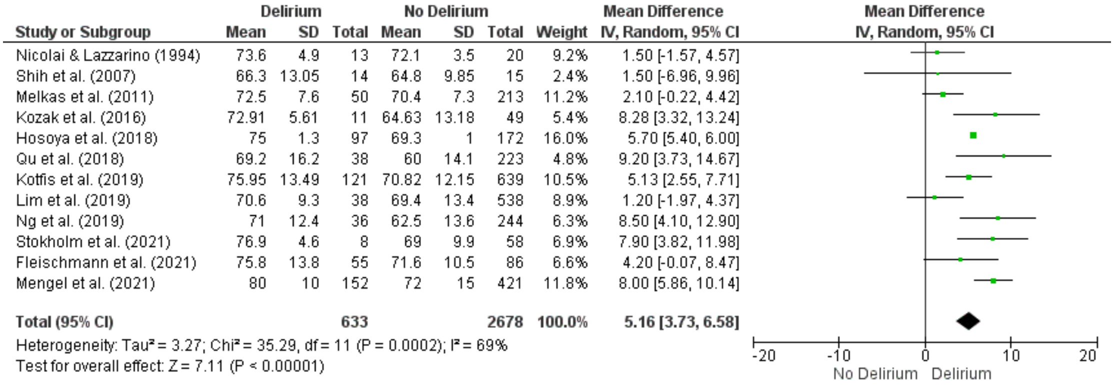

Figures S3a. The Forest Plot presenting the results from meta-analysis of age and PSD in stroke/TIA subgroup

	Delirium		No Delirium			Odds Ratio
Study or Subgroup	Events	Total	Events	Total	Weight	IV, Fixed, 95% CI
Melkas et al. (2011)	12	50	37	213	16.5%	1.50 [0.72, 3.15]
Kozak et al. (2016)	4	11	10	49	4.5%	2.23 [0.54, 9.14]
Kotfis et al. (2018)	83	172	210	829	79.0%	2.75 [1.96, 3.85]
**Total (95% CI)**		**233**		**1091**	**100.0%**	**2.47 [1.83, 3.33]**
Total events	99		257			

Heterogeneity: Chi² = 2.14, df = 2 (P = 0.34); I² = 7%
Test for overall effect: Z = 5.89 (P < 0.00001)

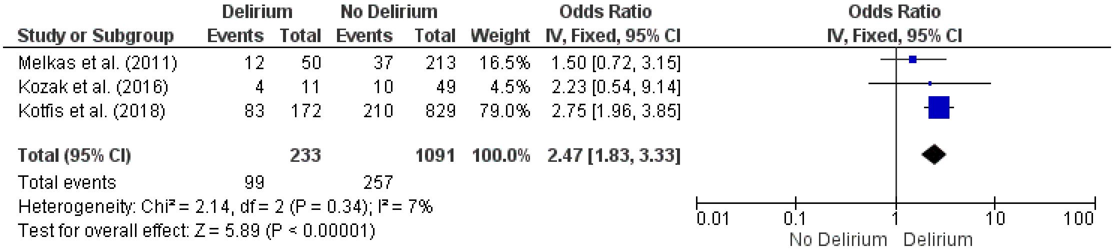

Figures S3b. The Forest Plot presenting the results from meta-analysis of AF and PSD in stroke/TIA subgroup

Study or Subgroup	Delirium Events	Delirium Total	No Delirium Events	No Delirium Total	Weight	Odds Ratio IV, Fixed, 95% CI
Qu et al. (2018)	14	38	32	223	11.0%	3.48 [1.63, 7.43]
Kotfis et al. (2018)	50	172	172	829	46.4%	1.57 [1.08, 2.27]
Ng et al. (2019)	9	36	54	244	9.6%	1.17 [0.52, 2.64]
Guldof et al. (2021)	54	201	48	313	33.0%	2.03 [1.31, 3.14]
**Total (95% CI)**		**447**		**1609**	**100.0%**	**1.81 [1.41, 2.33]**
Total events	127		306			

Heterogeneity: Chi² = 4.81, df = 3 (P = 0.19); I² = 38%
Test for overall effect: Z = 4.63 (P < 0.00001)

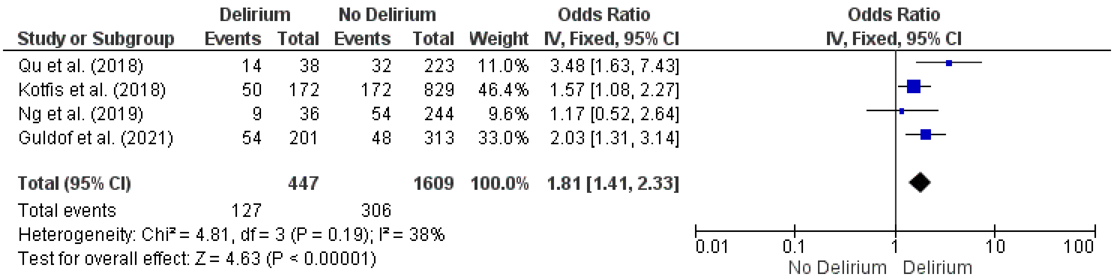

Figures S3c. The Forest Plot presenting the results from meta-analysis of prior stroke and PSD in stroke/TIA subgroup

Study or Subgroup	Delirium Events	Delirium Total	No Delirium Events	No Delirium Total	Weight	Odds Ratio IV, Random, 95% CI
Melkas et al. (2011)	8	50	40	213	18.9%	0.82 [0.36, 1.89]
Kozak et al. (2016)	12	49	6	11	10.2%	0.27 [0.07, 1.05]
Kotfis et al. (2018)	60	172	198	829	32.8%	1.71 [1.20, 2.43]
Ng et al. (2019)	8	36	40	244	18.3%	1.46 [0.62, 3.43]
Pendlebury et al. (2022)	14	67	16	127	19.9%	1.83 [0.83, 4.03]
**Total (95% CI)**		**374**		**1424**	**100.0%**	**1.22 [0.74, 2.01]**
Total events	102		300			

Heterogeneity: Tau² = 0.17; Chi² = 8.86, df = 4 (P = 0.06); I² = 55%
Test for overall effect: Z = 0.76 (P = 0.45)

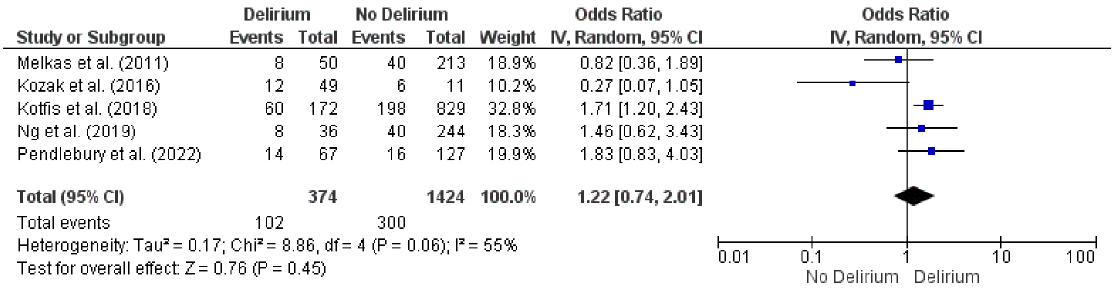

Figures S3d. The Forest Plot presenting the results from meta-analysis of CAD and PSD in stroke/TIA subgroup

Study or Subgroup	Delirium Events	Delirium Total	No Delirium Events	No Delirium Total	Weight	Odds Ratio IV, Fixed, 95% CI
Melkas et al. (2011)	10	50	41	213	20.5%	1.05 [0.48, 2.27]
Kozak et al. (2016)	3	11	4	49	4.4%	4.22 [0.79, 22.53]
Kotfis et al. (2019)	51	121	156	639	75.1%	2.26 [1.51, 3.38]
**Total (95% CI)**		**182**		**901**	**100.0%**	**1.98 [1.40, 2.81]**
Total events	64		201			

Heterogeneity: Chi² = 3.79, df = 2 (P = 0.15); I² = 47%
Test for overall effect: Z = 3.83 (P = 0.0001)

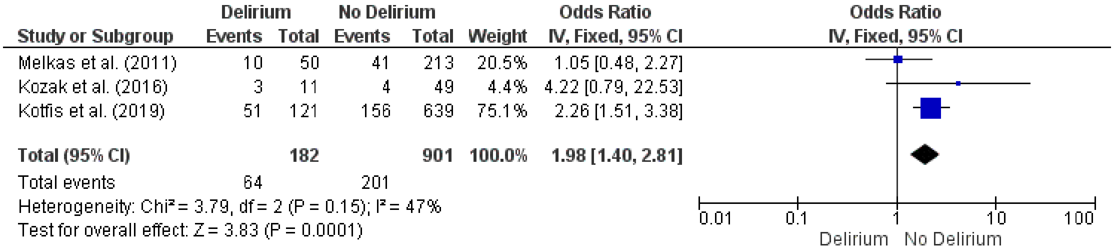

Figures S3e. The Forest Plot presenting the results from meta-analysis of HF and PSD in stroke/TIA subgroup

	Delirium		No Delirium			Odds Ratio	Odds Ratio
Study or Subgroup	Events	Total	Events	Total	Weight	IV, Fixed, 95% CI	IV, Fixed, 95% CI
Melkas et al. (2011)	5	50	24	213	8.5%	0.88 [0.32, 2.42]	
Kotfis et al. (2018)	106	172	348	829	77.4%	2.22 [1.59, 3.11]	
Pendlebury et al. (2022)	14	67	16	127	14.1%	1.83 [0.83, 4.03]	
**Total (95% CI)**		**289**		**1169**	**100.0%**	**2.00 [1.48, 2.68]**	
Total events	125		388				

Heterogeneity: Chi² = 2.95, df = 2 (P = 0.23); I² = 32%
Test for overall effect: Z = 4.57 (P < 0.00001)

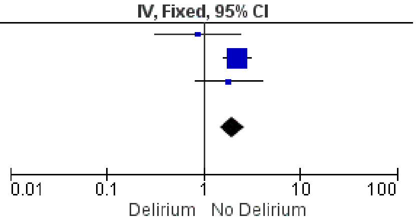

Figures S3f. The Forest Plot presenting the results from meta-analysis of PVD and PSD in stroke/TIA subgroup
