# Supplementary material for: The Role of Vascular Risk Factors in Post-Stroke Delirium: A Systematic Review and Meta-Analysis
Source: J Clin Med. 2022 Oct 1;11(19):5835. doi: 10.3390/jcm11195835 (PMC9571874; doi:10.3390/jcm11195835)
Supplement: Supplementary file 1 [file jcm-11-05835-s001.zip › Figures S4a to S4d.pdf]

Figures S4a to S4d. The Forest Plots presenting the results from meta-analysis of age, AF, prior stroke, and CAD in ICH and/or SAH subgroup

Study or Subgroup	Delirium Mean	Delirium SD	Delirium Total	No Delirium Mean	No Delirium SD	No Delirium Total	Weight	Mean Difference IV, Fixed, 95% CI
Naidech et al. (2013)	63.9	12.1	31	61.7	14.6	67	10.2%	2.20 [-3.31, 7.71]
Sauvigny et al. (2019)	56.6	13.4	78	53.8	13.4	134	22.0%	2.80 [-0.94, 6.54]
Reimann et al. (2021)	56	14	65	56	14	162	19.0%	0.00 [-4.03, 4.03]
Reznik et al. (2022)	72.1	15.5	348	68.3	15.2	242	48.8%	3.80 [1.29, 6.31]
**Total (95% CI)**			**522**			**605**	**100.0%**	**2.70 [0.94, 4.45]**

Heterogeneity: Chi² = 2.50, df = 3 (P = 0.48); I² = 0%
Test for overall effect: Z = 3.01 (P = 0.003)

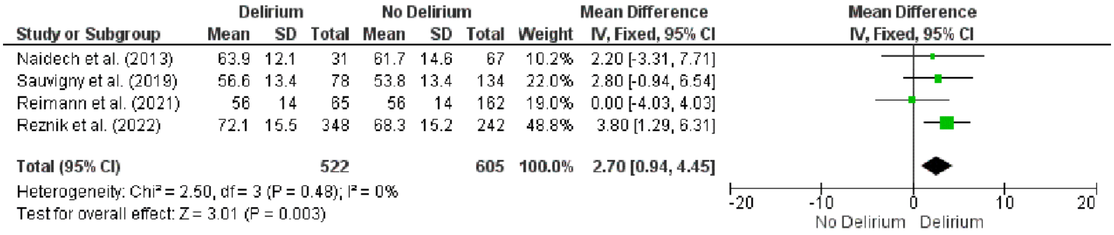

Figures S4a. The Forest Plot presenting the results from meta-analysis of age and PSD in ICH and/or SAH subgroup

Study or Subgroup	Delirium Events	Delirium Total	No Delirium Events	No Delirium Total	Weight	Odds Ratio IV, Fixed, 95% CI
Marrama et al. (2021)	9	74	29	174	18.5%	0.69 [0.31, 1.55]
Reznik et al. (2022)	73	348	66	242	81.5%	0.71 [0.48, 1.04]
**Total (95% CI)**		**422**		**416**	**100.0%**	**0.70 [0.50, 1.00]**
Total events	82		95			

Heterogeneity: Chi² = 0.00, df = 1 (P = 0.96); I² = 0%
Test for overall effect: Z = 1.98 (P = 0.05)

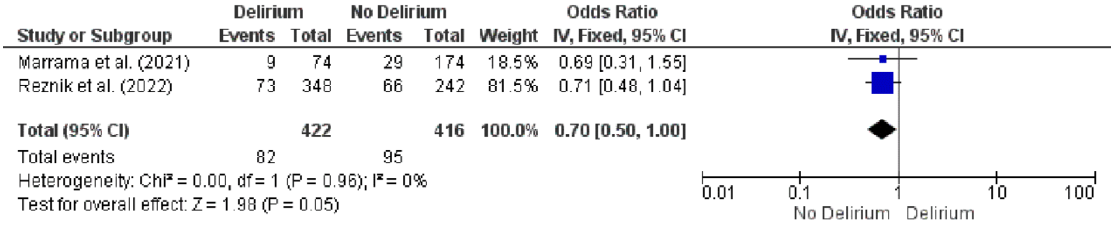

Figures S4b. The Forest Plot presenting the results from meta-analysis of AF and PSD in in ICH and/or SAH subgroup

Study or Subgroup	Delirium Events	Delirium Total	No Delirium Events	No Delirium Total	Weight	Odds Ratio IV, Fixed, 95% CI
Marrama et al. (2021)	16	74	28	174	27.6%	1.44 [0.72, 2.85]
Reznik et al. (2022)	86	348	38	242	72.4%	1.76 [1.15, 2.69]
**Total (95% CI)**		**422**		**416**	**100.0%**	**1.67 [1.16, 2.39]**
Total events	102		66			

Heterogeneity: Chi² = 0.24, df = 1 (P = 0.62); I² = 0%
Test for overall effect: Z = 2.78 (P = 0.005)

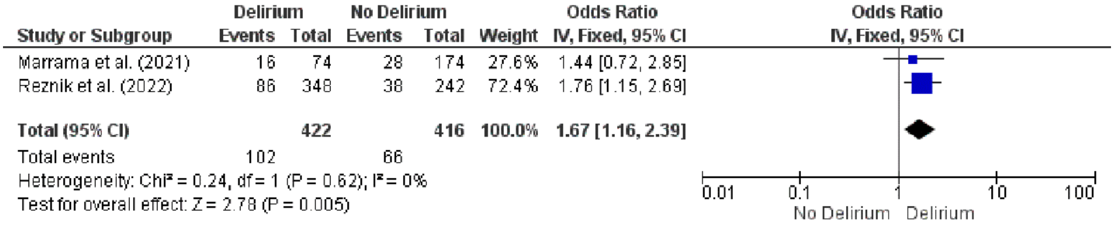

Figures S4c. The Forest Plot presenting the results from meta-analysis of prior stroke and PSD in ICH and/or SAH subgroup

Study or Subgroup	Delirium Events	Delirium Total	No Delirium Events	No Delirium Total	Weight	Odds Ratio IV, Fixed, 95% CI
Marrama et al. (2021)	17	74	28	174	34.7%	1.56 [0.79, 3.06]
Reznik et al. (2022)	66	348	25	242	65.3%	2.03 [1.24, 3.33]
**Total (95% CI)**		**422**		**416**	**100.0%**	**1.85 [1.24, 2.76]**
Total events	83		53			

Heterogeneity: Chi² = 0.39, df = 1 (P = 0.53); I² = 0%
Test for overall effect: Z = 3.03 (P = 0.002)

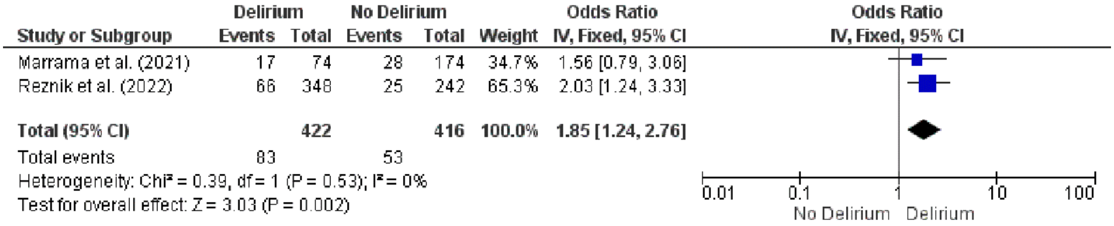

Figures S4d. The Forest Plot presenting the results from meta-analysis of CAD and PSD in ICH and/or SAH subgroup
