# Supplementary material for: The Role of Vascular Risk Factors in Post-Stroke Delirium: A Systematic Review and Meta-Analysis
Source: J Clin Med. 2022 Oct 1;11(19):5835. doi: 10.3390/jcm11195835 (PMC9571874; doi:10.3390/jcm11195835)
Supplement: Supplementary file 1 [file jcm-11-05835-s001.zip › Table S1.pdf]

Table S1. Quality evaluation according to the Newcastle-Ottawa Scale (NOS – for case-control and cohort studies) and modified NOS (for cross-sectional studies)

Study	Selection	Comparability	Exposure	NOS score
Gustafson et al. (1991)	***	**	**	7/9
Nicolai & Lazzarino (1994)	***	*	*	5/9
Henon et al. (1999)	****	**	***	9/9
Caerio et al. (2004)	***	**	**	7/9
Sheng et al. (2006)	***	**	***	8/9
Shih et al. (2007)	*		*	2/9
McManus et al. (2009)	***	**	***	8/9
Dahl et al. (2010)	***	**	**	7/9
Melkas et al. (2011)	****	**	**	8/9
Oldenbeuving et al. (2011)	****	**	***	9/9
Kostalova et al. (2012)	***	**	**	7/9
Kara et al. (2013)	***		**	5/9
Naidech et al. (2013)	****	**	***	9/9
Miu & Yeung (2013)	****	**	**	8/9
Turco et al. (2013)	***		***	6/9
Kozak et al. (2016)	**		**	4/9
Alvarez-Perez et al. (2017)	**	**	**	6/9
Ojagbemi et al. (2017)	***	**	**	7/9

Qu et al. (2018)	***	**	**	7/9
Hosoya et al. (2018)	***	**	**	7/9
Kotfis et al. (2018, 2019)	**	**	**	6/9
Lim et al. (2019)	***	**	**	7/9
Pasinska et. al (2018, 2019a, & 2019b), Czyzycki et al. (2021)	****	**	**	8/9
Aizen et al. (2019)	**		**	4/9
Zaitun et al. (2019)	*		**	3/9
Ng et al. (2019)	***	**	**	7/9
Sauvigny et al. (2019)	**	**	***	7/9
Shaw et al. (2019)	****	**	**	8/9
Rollo et al. (2021)	***	**	**	7/9
Reimann et al. (2021)	***	**	**	7/9
Nakamizo et al. (2021)	**	**	***	7/9
Guldof et al. (2021)	****	**	***	9/9
Fialho Silva et al. (2021)	****		***	7/9
Marrama et al. (2021)	****	**	**	8/9
Stokholm et al. (2021)	*		**	3/9
Zisper et al. (2021)	***	**	***	8/9
Fleischmann et al. (2021)	***	**	***	8/9
Mengel et al. (2021)	***		***	6/9

Reznik et al. (2020, 2022) ^	***	**	***	8/9
Pendlebury et al. (2022)	***	**	**	7/9

The number of * corresponds to the number of items assessed positively in each category
^Data from one of the studies are presented
