# Supplementary material for: The Role of Vascular Risk Factors in Post-Stroke Delirium: A Systematic Review and Meta-Analysis
Source: J Clin Med. 2022 Oct 1;11(19):5835. doi: 10.3390/jcm11195835 (PMC9571874; doi:10.3390/jcm11195835)
Supplement: Supplementary file 1 [file jcm-11-05835-s001.zip › Table S2.pdf]

Table 1. Characteristics of the included studies

Author (Year)	Ethnicity/Location	Phenotypes	Sample			Delirium	
			N	Mean Age, (SD) (y)	Female/Male	Assessment	Time
Gustafson et al. (1991)	Sweden	CI, TIA, ICH	145	73^	55/90	DSM-III-R	Within 7 days of admission
Nicolai & Lazzarino (1994)	Italy	PCA infraction	33	Confusion: 73.6 (4.9) No Confusion: 72.1 (3.5)	5/8 in PCA cases	Clinical/MMSE	NA
Henon et al. (1999)	France	CI, hemorrhage	202	75^	105/97	DSM-IV, DRS	After admission in SI, during hospitalization
Caerio et al. (2004)	Portugal	SAH, ICH, CI	218	57.3 (13)	88/130	DSM-IV, DRS	Within 4 days of admission
Sheng et al. (2006)	Australia	CI, ICH	156	79.2 (6.7)	73/83	DSM-IV	Within 3 days post-stroke
Shih et al. (2007)	Taiwan	PCA infraction	29	65.55	4/25	NA	At admission
McManus et al. (2009)	UK	CI, ICH	82	66.4 (15.9)	31/51	CAM	Within 4 days of admission
Dahl et al. (2010)	Norway	CI, hemorrhage	188	73	76/102	CAM, DSM-IV	Within first week of admission
Melkas et al. (2011)	Finland	Acute IS	263	70.8 (7.4)	128/135	DSM-IV	First 7 days after stroke
Oldenbeuving et al. (2011)	The Netherlands	IS, hemorrhage	527	72	239/288	CAM, DRS	Between days 2 and 4 after admission, and a second time between days 5 and 7
Kostalova et al. (2012)	Czech	CI, ISH	100	73.5 (11.5)	46/54	CAM-ICU, DSM-IV	Within 24 hours of stroke onset
Kara et al. (2013)	Turkey	IS, hemorrhage	150	Delirium: 68.0 (1.87) No Delirium: 61.2 (1.29)	45/105	DSM-IV, DRS	First five days
Naidech et al. (2013)	USA	ICH	98	Delirium: 63.9 (12.1) No Delirium: 61.7 (14.6)	NA	CAM-ICU	Within 28 days
Miu & Yeung (2013)	Hong Kong	CI, hemorrhage	314	72.9 (10.3)	151/163	CAM	Day 1-5
Turco et al. (2013)	Italy	IS, hemorrhage	176	81.7 (6.4)	118/58	CAM, DSM-IV-TR	At admission & at discharge

Kozak et al. (2016)	Turkey	IS	60	66.15 (12.53)	31/29	DSM-V, DRS	During hospitalization
Alvarez-Perez et al. (2017)	Portugal	Acute IS, hemorrhage	1072	68^	507/565	DSM-V	During hospitalization
Ojagbemi et al. (2017)	Nigeria	IS, hemorrhage	99	61.1 (12.9)	47/52	CAM, DRS	First 7 days of admission
Qu et al. (2018)	China	Acute IS	261	61.3 (14.8)	77/184	CAM	Within first week after admission
Hosoya et al. (2018)	Japan	CI, ICH, SAH	269	Delirium: 75 (1.3) No Delirium: 69.3 (1.0)	123/146	ICDSC	NA
Kotfis et al. (2018, 2019)*	Poland	Acute IS	1001	71^	478/523	CAM-ICU, DSM-V	Within 24 hours of admission
Lim et al. (2019)	Republic of Korea	IS	576	65.2	209/367	CAM	During admission
Pasinska et. al (2018, 2019a, & 2019b), Czyzycki et al. (2021)*	Poland	Acute IS, TIA, hemorrhage	750	71.75 (13.13)	398/352	bCAM, CAM-ICU, DSM-V	Between admission and 7th day
Aizen et al. (2019)	Israel	CI, ICH	110	80.2 (8)	57/53	CAM	From day 2 after admission to discharge
Zaitun et al. (2019)	Egypt	CI, ICH, SAH	74	60.7 (11.5)	34/40	DSM-IV	Within 3 days after stroke
Ng et al. (2019)	Malaysia	IS	280	63.6 (13.7)	94/186	CAM	Within 7 days of stroke
Sauvigny et al. (2019)	Germany	SAH	212	54.9 (13.4)	134/78	RASS	During the clinical course
Shaw et al. (2019)	Scotland	Stroke	395	Delirium: 79^ No Delirium: 65^	174/221	DSM-V, 4AT	Within 5 days of admission
Rollo et al. (2021)	Italy	IS, hemorrhage	120	71.8 (12.6)	48/72	CAM-ICU, RASS	Baseline and repeated within 72 h from admission
Reimann et al. (2021)	Austria	SAH	227	56 (14)	137/90	ICDSC, RASS	First 30 days after admission
Nakamizo et al. (2021)	Japan	IS, hemorrhage	387	75^	170/217	ICDSC	For 5 days from admission

Guldof et al. (2021)	Belgium	AIS	514	Delirium: 82^<br>No Delirium: 71^	237/277	DSM-V	During the first 7 days of admission
Fialho Silva et al. (2021)	Brazil	IS, hemorrhage	227	62.5 (13.5)	106/121	CAM-ICU, RASS	Admission until discharge
Marrama et al. (2021)	France	ICH	248	70.4 (12.7)	114/134	DRS, DSM-IV	First seven days of hospitalization
Stokholm et al. (2021)	Denmark	AIS	64	70 (9.8)	22/42	CAM	Hospitalization
Zisper et al. (2021)	Switzerland	IS, hemorrhage	567	Delirium: 76.0^<br>No Delirium: 70.0^	Delirium: male (115)	DOS, ICDSC, DSM-V	First 3 days of admission
Fleischmann et al. (2021)	Germany	IS, TIA	141	73.8 (10.4)	61/80	DMS-V , CAM, Nu-Desc, 4AT	Within 144hrs of admission
Mengel et al. (2021)	Germany	AIS, TIA	573	74 (14)	264/309	ICDSC, RASS, DSM-V	Within 90hrs of admission
Reznik et al. (2020, 2022) *	USA	ICH	590	70.5 (15.5)	181/309	DSM-V	During hospitalization
Pendlebury et al. (2022)	UK	Stroke, TIA	194	79.5 (11.2)	94/100	DSM-IV, CAM, AMTS	Admission

CI, cerebral infraction; TIA, transient ischemic attack; ICH, intracerebral hemorrhage; DSM, Diagnostic and Statistical Manual of Mental Disorders; MMSE, mini-mental state examination; DRS, Delirium Rating Scale; SI, stroke unit; SAH, subarachnoid hemorrhage; PCA, posterior cerebral artery; NA, non-available; UK, United Kingdom; CAM, Confusion Assessment Method; IS, ischemic stroke; bCAM, abbreviated version of Confusion Assessment Method; CAM-ICU, Intensive Care Units version of Confusion Assessment Method; ICDSC, Intensive Care Delirium Screening Checklist; RASS, Richmond Agitation and Sedation Scale; DOS, Delirium Observation Screening Scale; 4AT, rapid assessment test for delirium; Nu-Desc, Nursing Delirium Screening Scale; AMTS, abbreviated mental test score

*Data from one of the studies are presented

^median
